# Molecular Structure, In Vitro Anticancer Study and Molecular Docking of New Phosphate Derivatives of Betulin

**DOI:** 10.3390/molecules26030737

**Published:** 2021-01-31

**Authors:** Elwira Chrobak, Maria Jastrzębska, Ewa Bębenek, Monika Kadela-Tomanek, Krzysztof Marciniec, Małgorzata Latocha, Roman Wrzalik, Joachim Kusz, Stanisław Boryczka

**Affiliations:** 1 Department of Organic Chemistry, Faculty of Pharmaceutical Sciences in Sosnowiec, Medical University of Silesia, Katowice, 4 Jagiellońska Str., 41-200 Sosnowiec, Poland; ebebenek@sum.edu.pl (E.B.); mkadela@sum.edu.pl (M.K.-T.); kmarciniec@sum.edu.pl (K.M.); boryczka@sum.edu.pl (S.B.); 2 Silesian Center for Education and Interdisciplinary Research, Institute of Physics, University of Silesia, 75Pułku Piechoty 1a, 41-500 Chorzów, Poland; maria.jastrzebska@us.edu.pl (M.J.); roman.wrzalik@us.edu.pl (R.W.); 3 Department of Cell Biology, Faculty of Pharmaceutical Sciences in Sosnowiec, Medical University of Silesia, Katowice, 8 Jedności Str., 41-200 Sosnowiec, Poland; mlatocha@sum.edu.pl; 4 Institute of Physics, University of Silesia, 75 Pułku Piechoty Str.1a, 41-500 Chorzów, Poland; joachim.kusz@us.edu.pl

**Keywords:** natural compounds, betulin, anticancer study, molecular docking, EGFR

## Abstract

A series of 30-diethylphosphate derivatives of betulin were synthesized and evaluated for their in vitro cytotoxic activity against human cancer cell lines, such as amelanotic melanoma (C-32), glioblastoma (SNB-19), and two lines of breast cancer (T47D, MDA-MB-231). The molecular structure and activities of the new compounds were also compared with their 29-phosphonate analogs. Compounds **7a** and **7b** showed the highest activity against C-32 and SNB-19 cell lines. The IC_50_ values for **7a** were 2.15 and 0.91 μM, and, for **7b,** they were 0.76 and 0.8 μM for the C-32 and SNB-19 lines, respectively. The most potent compounds, **7a** and **7b**, were tested for their effects on markers of apoptosis, such as H3, TP53, BAX, and BCL-2. For the whole series of phosphate derivatives, a lipophilicity study was performed, and the ADME parameters were calculated. The most active products were docked to the active site of the EGFR protein. The relative binding affinity of selected phosphate betulin derivatives toward EGFR was compared with standard erlotinib on the basis of ChemScore and K_DEEP_ score. Positively, all derivatives docked inside the cavity and showed significant interactions. Moreover, a molecular dynamics study also reveals that ligands **7a**,**b** form stable complexes and the plateau phase started after 7 ns.

## 1. Introduction

Cancer is a large group of diseases in which the mechanisms of cell growth control have been lost. Today, it is one of the leading causes of death in people all over the world (approximately 30% of all premature deaths among adults aged 30–69 years not caused by infectious diseases) [1]. Chemotherapy with synthetic drugs is burdened with numerous side effects. Therefore, substances of natural origin are being considered a potential source of new anticancer drugs.

Triterpenes are an important class of compounds synthesized by various plants, which show a broad spectrum of biological activity [2,3]. Betulin and betulinic acid are among the triterpenes of the lupane type. The structure of these compounds contains substituents such as isopropenyl moiety and hydroxyl and/or carboxyl groups that can be easily functionalized. This allows for the continuous expansion of the library of derivatives (terpenoids), and among these compounds, new substances with potential biological activity are sought. In the group of semi-synthetic derivatives of betulin, numerous compounds resulting from modification in the isopropenyl group have also been described. The products obtained in this way were tested for various activities, including, for example, anti-inflammatory, antiviral, and anticancer properties [4,5,6].

As a result of introducing a function containing phosphorus atoms into isopropenyl moiety of betulin diesters, compounds with antitumor (diethylphosphonate derivatives) and antischistosomal (triphenylphosphonium derivatives) activity were obtained [7,8]. Anti-HIV-1 studies performed with the series of phosphorus analogs of bevirimat showed that the 30-diethylphosphate derivative has comparable activity to bevirimat, but with higher selectivity [9].

The carbon–carbon triple bond occurs in drugs used in the pharmacotherapy of various diseases and is still the subject of many research studies related to the development of new pharmaceuticals [10]. Examples include the antiretroviral efavirenz, the contraceptive norethynodrel, oxybutynin for the treatment of overactive bladder, pargyline with the antidepressant effect, the antitumor erlotinib, and the antifungal terbinafine [11].

**Figure 1:**
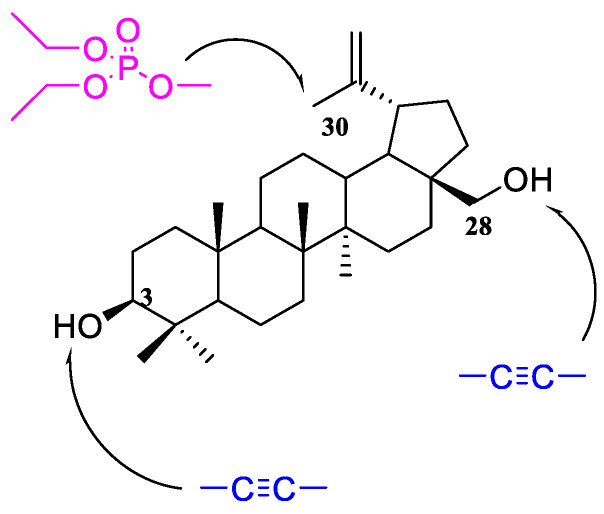
Schematic structure of target compounds.

The literature review shows that the presence of an alkynyl moiety in a compound molecule may favorably influence its antitumor activity [12,13,14]. This is also confirmed by the studies conducted earlier by our team [15,16,17,18,19]. It, therefore, seemed interesting to obtain new betulin derivatives containing diethylphosphate group in position C30, and an alkynyl substituent in position C3 and/or C28 (Figure 1).

The aim of the present study was the synthesis of new betulin derivatives, analysis of the structure of these compounds, and evaluation of their in vitro cytotoxic activity. Human glioma (SNB-19), melanoma (C-32), and two breast cancer (T47D and MDA-MB-231) cell lines were used in the research. In addition, in silico techniques such as ADMET parameter prediction and molecular docking were used to more fully characterize novel substances with potential anticancer effects. The comparison of the structure and activity of phosphonate and phosphate derivatives carried out in the paper was to answer the question of how the replacement of the phosphonate with the phosphate group in the isopropenyl substituent affects the cytotoxic activity of betulin alkynyl derivative.

## 2. Results and Discussion

### 2.1. Synthesis

In order to modify the betulin molecule or its derivatives at position C30, first the functionalization of the allylic hydrogen is carried out, introducing a substituent of greater reactivity into it. The most common method for this purpose is bromination at the allyl position with *N*-bromosuccinimide (NBS) [20]. The 30-bromo- derivatives thus obtained are a convenient substrate for further transformations. They have been used in reactions with thiolates, aliphatic and alicyclic alcohols, potassium isocyanate, sodium azide, acid anhydrides, dimethylaminopyridine (DMAP), triphenylphosphine, and trialkyl phosphite. As a result of these reactions, new compounds were obtained that were evaluated for biological activity [6,7,8,20].

Another route for the preparation of the 30-substituted derivatives is the introduction of a hydroxyl group by oxidation of the isopropenyl moiety with m-chloroperoxybenzoic acid (*m*-CPBA) [21].

**Scheme 1:**
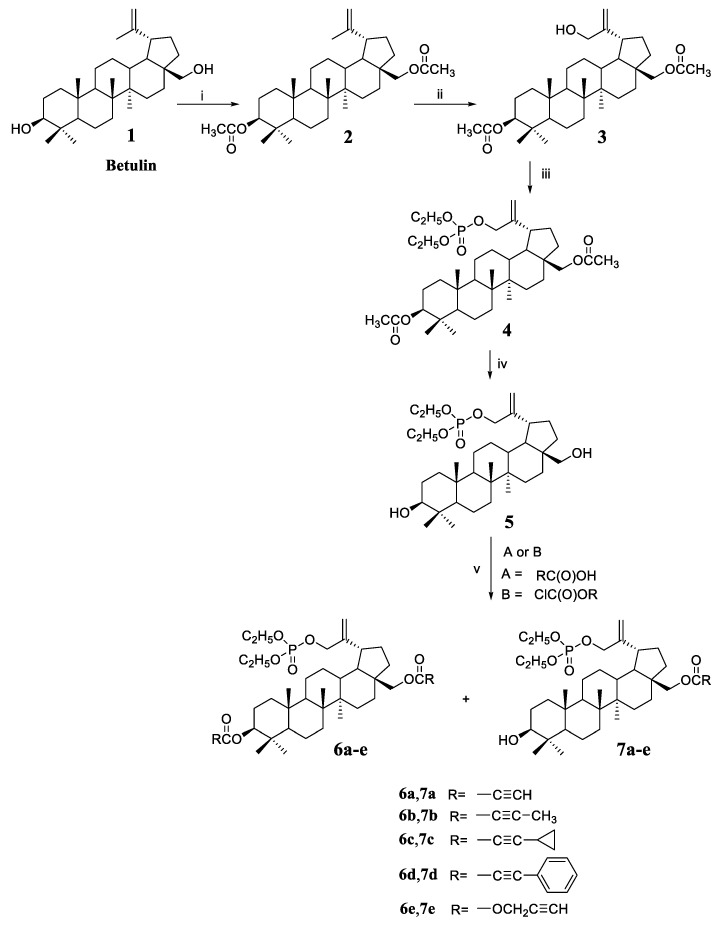
Synthesis of 30-diethoxyphosphoryloxybetulin **5** and its alkynyl derivatives **6a**–**6e** and **7a**–**7e**. Reaction conditions: (i) (CH_3_CO)_2_O, pyridine, room temp.; (ii) *m*-CPBA, CHCl_3_, reaction at boiling temp.; (iii) (C_2_H_5_O)_2_P(O)Cl, DMAP, pyridine, room temp.; (iv) NaOH, THF/MeOH/H_2_O, room temp.; (v) for A—DCC, DMAP, CH_2_Cl_2_, from −10 to room temperature, for B—pyridine, benzene, from −5 to room temp.

3,28-Diacetyl-30-hydroxybetulin **3** (Scheme 1) was used as the starting compound for this work. This compound has a free hydroxyl group at position 30, which can be converted into a phosphate substituent (derivative **4**). The phosphorylation reaction was carried out with diethyl chlorophosphate in a pyridine solution. The next step was the hydrolysis of acetyl groups at C3 and C28 leading to deprotection of the hydroxyl groups, which enabled their further functionalization. Hydrolysis of the acetyl group is most often performed in an alkaline medium [22]. First, the substituent is unblocked at the C28 position (primary hydroxyl group), then at C3 (secondary hydroxyl group). Unfortunately, under these conditions, the C-O-P(O)(OEt)_2_ ester bond can also be hydrolyzed. Hydrolysis was carried out with sodium hydroxide in a water/tetrahydrofuran/methanol mixture in a volume ratio of 1:2:1. The course of the reaction was monitored by thin layer chromatography, and thus the optimal duration of 5 h was determined. Analysis of the spectra of the hydrolysis product **5** showed that no removal of the phosphate group had occurred under the reaction conditions used. This fact is confirmed by the presence in the ^31^P spectrum of the signal of the phosphorus atom at −0.91 ppm.

As already mentioned, the introduction of a substituent containing a triple bond to the betulin molecule may have a beneficial effect on the cytotoxic activity of the compound. Reactions of 30-diethoxyphosphoryloxybetulin **5** with four alkynyl acids were performed using free hydroxyl groups. Acids with different structures were selected for the synthesis, i.e., propiolic acid with a terminal triple bond and acids with a C≡C bond in the middle of the carbon chain, i.e., 2-butynoic, cyclopropylpropiolic, and phenylpropiolic acid.

Reactions with acids were performed using the Steglich method, which is suitable for substrates requiring mild conditions and a non-acidic environment [23]. The use of a 17% excess of alkynyl acid guarantees complete conversion of the hydroxyl group at the C28 position of the starting substrate **5**, leading to the formation of monosubstituted derivatives **7a**–**7d** that were isolated in 71–82% yields. Additionally, small amounts of diesters **6a**–**6d** are formed in the reaction that were obtained in 5–12% yields.

The reaction of **5** with propargyl chloroformate was carried out in benzene in the presence of pyridine to give the alkynyloxycarbonyl derivatives **7e** and **6e** in yields 60% and 15%, respectively.

All products were purified by column chromatography. The structures of compounds **3**–**5**, **6a**–**6e**, and **7a**–**e** were confirmed based on the analysis of ^1^H, ^13^C, and ^31^P NMR spectra and the mass spectrometry method (HR-MS).

**Scheme 2:**
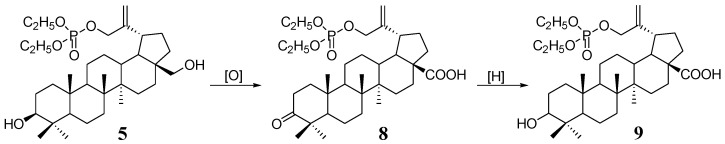
Synthesis of 30-phosphate derivatives of betulonic (**8**) and betulinic acid (**9**) from 30-diethoxyphosphoryloxybetulin (**5**).

In general, there is a wealth of information about the antitumor activity of betulinic acid. Taking this into account, phosphate derivatives of betulonic **8** and betulinic acid **9** were also included in this study (Scheme 2). In our earlier work, we described the synthesis and testing of anti-HIV-1 activity of these compounds, but so far they have not been tested as anticancer agents [9].

### 2.2. Structural Studies

One of the steps in the synthesis of derivatives containing a phosphate group at the C30 position is the hydrolysis of acetyl groups at the C3 and C28 positions. This reaction is carried out in an alkaline medium. As previously described, for 3,28-diacetyl-30-diethylphosphonobetulin, this reaction proceeds with allyl–vinyl isomerization in the isopropenyl group [7]. The compound **4** contains a phosphate group in the allyl position (C30), so the substituent is connected via an oxygen atom. Based on ^1^H and ^31^P NMR spectra of the hydrolysis product (compound **5**), isomerization was not observed. The structure of product **5** was confirmed based on the analysis of ^31^P NMR spectra (singlet at δ −0.91 ppm), and also ^1^H NMR (signals δ = 4.15 ppm and δ = 1.37 ppm), which prove the presence of the ethyl groups of the phosphate substituent (Figure S2a,c). The position of the H3 proton signals (δ = 3.21 ppm) and H28 (δ = 3.80 ppm and δ = 3.32 ppm) corresponds to the chemical shift of these protons in the spectra of unsubstituted betulin containing free hydroxyl groups. In the ^13^C NMR spectrum of compound **5** (Figure S2b), the signals of the C3 and C28 carbon atoms are shifted by about 3 ppm (δ = 77.9 ppm and δ = 59.2 ppm, respectively) compared to the position in the substrate **4** spectrum (δ = 80.9 ppm and δ = 62.4 ppm) including acetyl groups (Figure S1b). This shift is due to the absence of the adjacent electron-accepting carbonyl group, which had a revealing effect.

Using the X-ray structural study of a single-crystal and Raman spectra, the structure of new 30-phosphate derivatives was analyzed. Additionally, a comparison was made with selected 29-phosphonate analogs in terms of the position of the double bond in the isopropenyl group.

#### 2.2.1. Raman Spectra

The experimental Raman spectra for phosphate and phosphonate derivatives were measured in the frequency range of 200–2000 cm^−1^ using a confocal alpha 300R (WITec, Germany) spectrometer.

**Figure 2:**
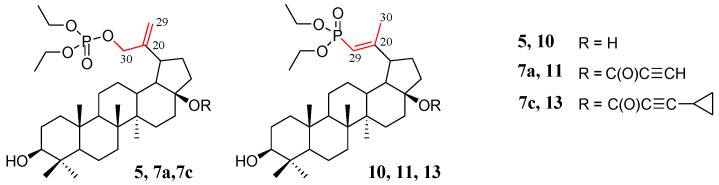
Structure of allyl substituted compounds **5**, **7a**, and **7c** (phosphate derivatives) and vinyl **10, 11,** and **13** (phosphonate derivatives).

Compounds for which experimental Raman spectra were made were grouped into three sets depending on the type of substituents, i.e., (**5**, **10**), (**7a**, **11**), and (**7c**, **13**) (Figure 2).

The two compounds in each set differ in the position of the C=C double bond and formation of the O=P-O-C (allyl substituted compounds **5**, **7a**, and **7c**) or O=P-C=C (vinyl substituted compounds **10**, **11**, and **13**) groups, respectively (Figure 2). In phosphate derivatives, this group is attached through an oxygen atom, and the distances of the phosphoryl group (P=O) from the C=C double bond are different.

Experimental frequencies and band assignments are collected in Table 1. The theoretical Raman spectra for selected compounds, i.e., **5** and **10**, were calculated using the DFT method (see Materials and Methods). In Table S1, the calculated band frequencies and assignments for **5** and **10** are collected and included in the Supplementary Materials.

The band assignment of experimental spectra was performed by comparison with those calculated as well as using the literature databases of the IR and Raman spectra [24,25].

Comparing the intensities and positions of the individual theoretical and experimental peaks (Table 1 and Table S1), we can notice quite significant differences, which, however, do not affect the quality of band assignment to individual vibrations. This difference is mainly due to the approximations of the calculation model (the size of the base wave functions and harmonic vibrations approximation) and the fact that the calculations were performed for a single molecule and the interactions of molecules were not taken into account (see Materials and Methods). Despite the limitations of the model, the spectral calculations provided helpful additional information for the interpretation of the experimental data.

**Figure 3:**
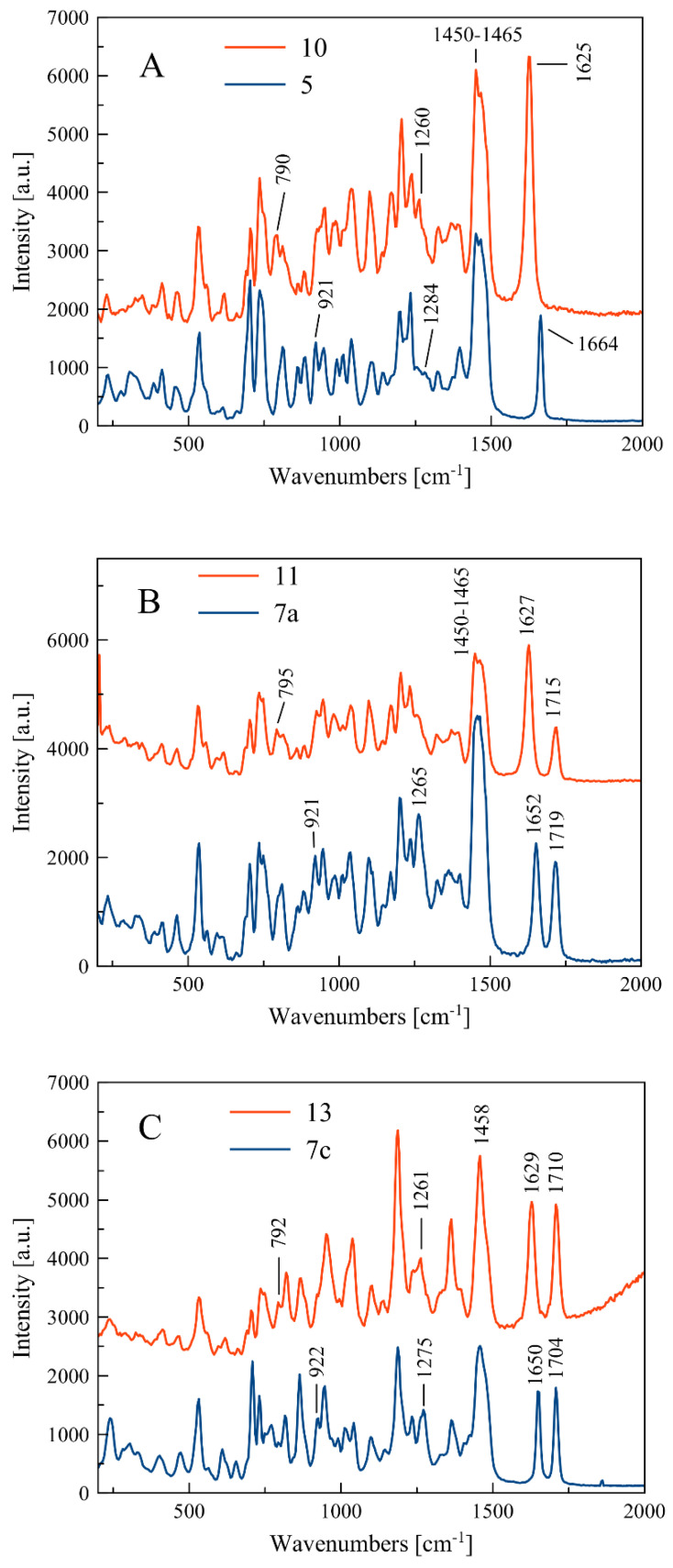
Experimental Raman spectra in the range 200–2000 cm^−1^ for three sets of compounds: (**5**, **10**) (**A**), (**7a**, **11**) (**B**), and (**7c**, **13**) (**C**).

Raman spectra of all investigated compounds show intense bands in the range of 1664–1625 cm^−1^ assigned to the stretching vibration of the C=C double bound (Figure 3). According to literature data, the C=C stretching band in alkyl-substituted ethylenes is usually located in the neighborhood of 1650 cm^−1^. The position of the C=C stretching vibration band is influenced by the adjacent functional groups, so in the molecule **5**, one of the carbon atoms is bound to two hydrogen atoms and the other one to hydrogen and the -CH_2_- group connected to the phosphate residue. In compound **10**, however, one carbon atom is connected to a hydrogen atom and a methyl group, and the other to a hydrogen atom and a phosphorus atom of the phosphonate group (Figure 2).

The frequencies of the individual C=C stretching bands νc=c for the studied compounds are summarized in Table 2.

Within each set of the compounds there is a band shift (Δνc=c)_exp_ due to different positions of the C=C bond in the side chain. For compounds **5** and **10**, the change in location of the C=C bond is accompanied by shortening of the bond length from 1.338(3) Å to 1.326(5) Å and causes the band shift towards larger wavelength by the value of (Δνc=c)_exp_ = 39 cm^−1^. The calculated band shift (Δνc=c)_cal_ for these compounds is much higher and equal to 68 cm^−1^ (see Table 2). For sets of compounds **7a**–**11** and **7c**–**13**, the bond shifts (Δνc=c)_exp_ are equal to 25 and 21 cm^−1^, respectively.

As shown in Table 1, the bands at 1284 and 1275 cm^−1^ relate to stretching vibration of the P-O bond in the O=P-O-C side chain, which appear in compounds **5** and **7c**. In turn, the P-C stretching vibration in the O=P-C=C side chain at 1260 and 1261 cm^−1^ can be observed for compounds **10** and **13**, respectively. It can be noticed that there are some deviations from this scheme, i.e., the P-O and P-C stretching bands do not appear clearly in the spectra of compounds **7a** and **11**, as shown in Figure 2.

Similarly, the bending vibrations of the P-O and P-C bonds are observed in ranges 921–926 and 790–795 cm^−1^, for **5**, **7a**, and **7c** and **10**, **11**, and **13**, respectively (see Table 1).

For compounds **7a**, **7c**, **11**, and **13**, the single peak of C=O stretching vibration in the range 1704–1719 cm^−1^ can be observed. The C-H bending vibration in the side chain and in the cycloalkane ring as well as the C-C cycloalkane ring vibrations appear mainly at low frequencies, e.g., about 700–500 cm^−1^, and they are confirmed by theoretical spectra (see Table S1).

#### 2.2.2. Crystal Structure

**Figure 4:**
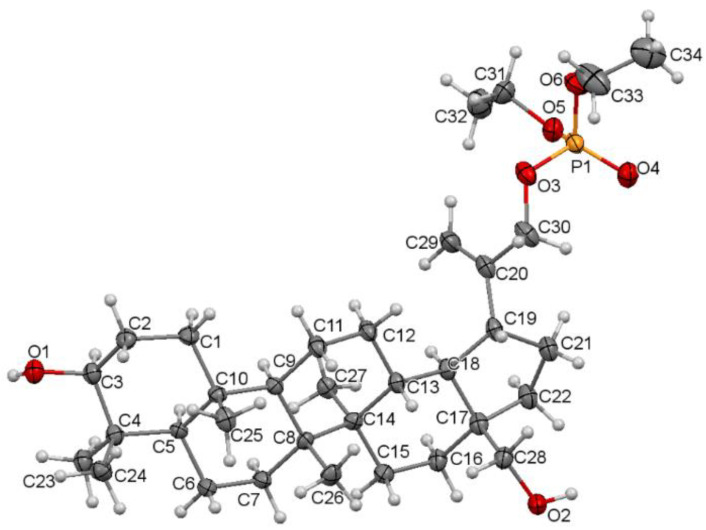
Molecular structure of the compound **5** showing the atom numbering scheme. Displacement ellipsoids are drawn at the 50% probability level.

The single crystal suitable for the X-ray diffraction analysis was grown by slow evaporation at room temperature technique from acetonitrile solution. The molecular structure of the compound **5** with atom numbering is shown in Figure 4. Additional information about the crystal can be found in Supplementary Materials, Tables S2 and S3.

Compound **5** crystallizes in a monoclinic P2_1_ space group. There are two molecules per asymmetric unit. The molecular structure of **5** is stabilized by hydrogen bonds as well as weak intermolecular C-H⋯O bonds (Table S4). In the pentacyclic scaffold of betulin, the six-membered rings adopt chair conformations and are joined similarly, as described previously for other betulin derivatives [7]. The five-membered ring assumes the conformation of the twisted envelope. Structure analysis confirms that in the derivative **5** the diethyl phosphate substituent is connected to the betulin molecule via an ester bond attached to a sp^3^ hybridized carbon (C30) atom. The length of the C20–C30 bond for compound **5** is 1.490 Å, and it is longer than the C20–C29 bond equal 1.326 Å (Table 3).

**Figure 5:**
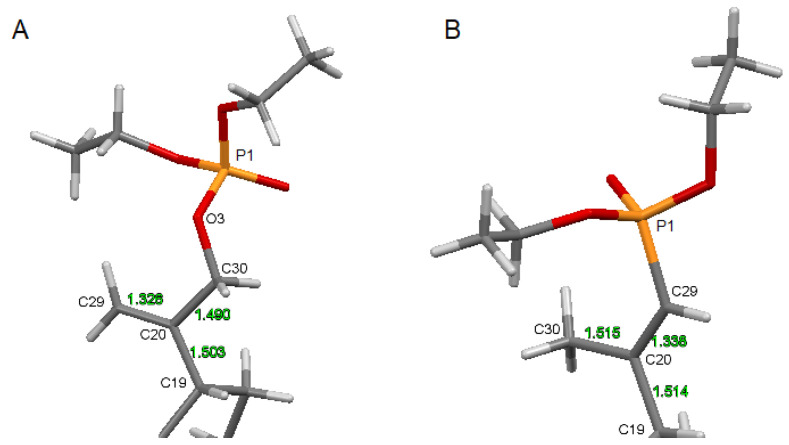
The structure of the substituted isopropenyl group: (**A**)—for compound **5**, (**B**)—for reference phosphonate derivative **13**.

The comparison of the structure of an isopropenyl group containing a phosphonate (**13**) and a phosphate (**5**) substituent is shown in Figure 5.

### 2.3. Anticancer Activity

The anticancer activity has been tested for various alkynyl derivatives of triterpenes, and the results obtained were promising [13,26,27]. On the other hand, the literature also contains information that the introduction of a dialkylphosphate group into a molecule of a known drug substance may improve its activity or even change it towards having an antitumor effect [28,29]. The combination of these two moieties in the betulin molecule could create a derivative with higher antitumor activity. Thus, the phosphate derivatives synthesized in this study **4**, **5**, **6a**–**6e**, and **7a**–**7e** were assessed for in vitro activity against four human tumor cell lines such as amelanotic melanoma (C-32), glioblastoma (SNB-19), and two lines of breast cancer (Luminal A class—T47D; Claudin—low class; a triple negative breast cancer—MDA-MB-231).

#### 2.3.1. Cell Viability Studies

Evaluation of the effect of new betulin derivatives on the viability of the cultured cells was performed using the WST-1(water soluble tetrazolium salt) assay. Betulin **1** and cisplatin were used as the reference compounds. The results, expressed as half the maximum inhibitory concentration (IC_50_), are shown in Table 4.

For all cell lines tested, the 3,28-diester derivatives **6a**-**e** were less active (IC_50_ range 4.65–31.5 μM) than the 28-monoester derivatives **7a**–**7e** (IC_50_ range 0.76–15.40 μM). Among the monoester compounds, **7b** and **7a** were the most active. They contain, respectively, 2-butynoyl and propynoyl substituents. Monoester derivatives of acids with a more extensive structure, **7c** (cyclopropylpropynoyl) and **7d** (phenylpropynoyl), were significantly less active, but their activity was higher compared to betulin **1**.

It can be seen that among the cell lines tested, cells of both breast tumors were characterized by the lowest sensitivity to alkynyl derivatives of betulin phosphate. The highest activity for the T47D (IC_50_ = 1.04 μM) and MDA-MB-231 (IC_50_ = 0.40 μM) lines was determined for the 30-phosphate of betulin **5**. In the group of phosphate derivatives, only the 28-propynoyl derivative **7a** showed a significant effect on T47D cells (IC_50_ = 2.61 μM). As previously described, compounds containing a phosphonate group at position C29, analogous betulin derivatives, were active against this cell line; however, the unsubstituted compound **10** (IC_50_ = 1.55 μM) was less active than the 28-propynoyl **11** (IC_50_ = 0.70 μM) analog [Table 4].

The C-32 and SNB-19 cells seem to be more sensitive to 30-phosphate betulin derivatives. Compound **7b** showed the strongest activity for both lines (IC_50_ = 0.76 and 0.8 μM, respectively).

The derivative **7a** with the terminal triple bond was slightly weaker in relation to SNB-19 cells (IC_50_ = 0.91 μM) and more than two times less active towards the C-32 line (IC_50_ = 2.15 μM). Among betulin 29-phosphonates, the activity of the corresponding propynoyl derivative **11** (IC_50_ = 0.6 μM) was significantly higher than that of the 2-butynoyl derivative **12** (IC_50_ = 7.70 μM). A meaningful difference in the cytotoxicity of 28-propargyloxycarbonyloxy derivatives was also observed. The structure–activity relationships observed in the 30-phosphate monoesters **7a**–**7e** indicate that the order of the substituents in the cytotoxic effect on the SNB-19 cell line is as follows: 2-butynoyl > propynoyl > propargyloxycarbonyl > 3-cyclopropyl-2-propynoyl > 3-phenyl-2-propynoyl. Comparing the activities of compound **7a** with those determined for 28-O-propynoylbetulin in relation to appropriate tumor cell lines (IC_50_ values 16.7, 4.2, and 110.8 μM for C-32, SNB-19, and T47D, respectively) [27], it seems that the introduction of a diethylphosphate substituent to the isopropenyl group may exert a beneficial effect on the cytotoxic activity of the obtained derivatives.

The phosphate derivatives of betulonic (**8**) and betulinic (**9**) acids showed no activity in relation to the examined breast cancer lines (T47D and MDA-MB-231). Activity against C-32 and SNB-19 cells determined for these compounds as well as the references (29 and 30 phosphonate derivatives and betulinic acid) are summarized in Table 5.

Initial studies of the anticancer activity of betulinic acid were focused on human melanoma cells, and then its effects on numerous other cancer cell lines, including glioblastoma, have been reported [30,31]. The evaluation of the activity of phosphorus derivatives of betulonic acid and betulinic acid, carried out in our study, showed that these compounds are much less active against C-32 melanoma cells (IC_50_ range 30.74–47.46 μM) than betulinic acid (IC_50_ = 8.63 μM; R=H, R_1_=OH). The tested acid derivatives showed a much greater effect on SNB-19 cells. The strongest activity (IC_50_ = 1.29 μM) was presented in the 30-phosphonate derivative of 3-acetylbetulinic acid (R=30-P(O)(OC_2_H_5_)_2_, R_1_=CH_3_C(O)O). The IC_50_ value determined for 30-phosphate of betulinic acid **9** was more than 4.5-fold higher.

#### 2.3.2. Analysis of the Expression of Genes Related to the Apoptosis Process

In the fight against cancer, it is especially important to understand the mechanisms involved in cell death. The effectiveness of anticancer therapy results not only from the inhibition of proliferation, but also from the ability to induce apoptosis in cancer cells. Apoptosis, also known as programmed cell death, is a fundamental physiological process that plays a key role in the development and maintenance of tissue and body homeostasis. A cell dying in the process of apoptosis undergoes both morphological changes (chromatin condensation, changes in the structure of the cell nucleus, and formation of apoptotic bodies) and biochemical changes (activation of many proteins classified as both transcription factors and enzymes and changes in the structure of the cell membrane) [32]. Changes in apoptotic cells are determined by the expression of relevant genes that are involved in this complex process and act both pro-and anti-apoptotically [33,34,35].

In our work, we assessed the influence of the tested compounds on the expression of selected genes related to apoptosis. The two most active derivatives **7a** and **7b** were selected for the study. We examined their influence on the transcriptional activity of genes encoding a proliferation marker (histone H3), a cell cycle regulator (p53), and proteins related to the mitochondrial apoptosis pathway (BCL-2 and BAX). The study was performed after 24-h exposure of the cultured cells to the test compounds (half of IC_50_).

The structural organization of DNA into chromatin is essential for maintaining cellular identity. In the S phase of the cell cycle, the chromatin structure is multiplied (related to, among others, the synthesis of histones), which is coupled with DNA replication. Histone H3, being one of the components of chromatin, undergoes several post-translational modifications, influencing gene expression. According to the literature data, the level of H3 mRNA quantified by QRT-PCR can serve as a marker in the assessment of proliferative activity and the development of brain glioma, for example [34,35].

**Figure 6:**
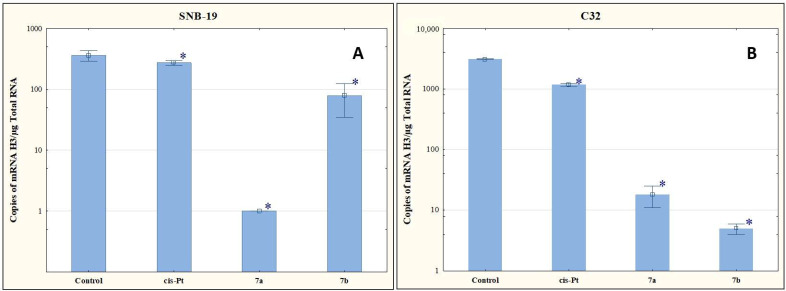
The effect of compounds **7a** and **7b** on transcriptional activity of H3 for SNB-19 (**A**) and C-32 (**B**) cells. Cells were exposed to compound **7a** and **7b** for 24 h at a concentration equal to half of IC_50_ determined for SNB-19 (0.3 and 0.25 µg/mL, respectively) and for C-32 (0.7 and 0.25 µg/mL, respectively). For the reference compound, cisplatin, the appropriate concentrations were 0.8 µg/mL (SNB-19 line) and 0.6 µg/mL (C32 line). * *p* < 0.05 compared with the untreated control.

In our study, compounds **7a** and **7b** reduced the number of mRNA copies of the histone H3 gene in the cells of both tumor lines compared to the level determined for the control (Figure 6). The antiproliferative effect of these compounds may therefore be due to the ability to reduce the histone H3 genes at the mRNA level.

Most cancers have a mutation in the TP53 suppressor gene, which encodes the P53 protein. This leads to an inhibition of cell apoptosis and tumor growth. In apoptosis induced by DNA damage, the p53 protein is involved in various activation steps in both the internal and external pathways. It is related to its function as a transcription factor of pro-apoptotic genes and its direct interaction with anti-apoptotic proteins, which results in their blockade [36].

**Figure 7:**
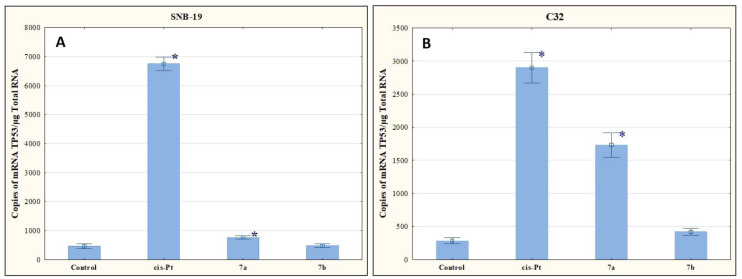
The effect of compounds **7a** and **7b** on transcriptional activity of TP53 for SNB-19 (**A**) and C-32 (**B**) cells. Cells were exposed to compound **7a** and **7b** for 24 h at a concentration equal to half of IC_50_ determined for SNB-19 (0.3 and 0.25 µg/mL, respectively) and for C-32 (0.7and 0.25 µg/mL, respectively). For the reference compound, cisplatin, the appropriate concentrations were 0.8 µg/mL (SNB-19 line) and 0.6 µg/mL (C32 line). * *p* < 0.05 compared with the untreated control.

The results of our research presented in Figure 7 indicate that the tested compounds increase the number of mRNA copies of TP53. Compound **7b** upregulated TP53 expression levels, but not as significantly as was observed for **7a**.

There is a strong genetic and biochemical link between the tumor suppressor p53 and the oncogene BCL-2 that is fundamental to cancer biology. The BCL-2 family of proteins includes both pro-apoptotic (e.g., BAX) and anti-apoptotic (e.g., BCL-2) proteins. The task of anti-apoptotic proteins from the BCL-2 family is to bind to the active form of BAX, which prevents it from penetrating into the outer membrane of the mitochondria and blocks apoptosis [33,37,38]. The effectiveness of anti-cancer therapies depends on the mechanisms of BCL-2/BAX-dependent cancer cell killing [38].

**Figure 8:**
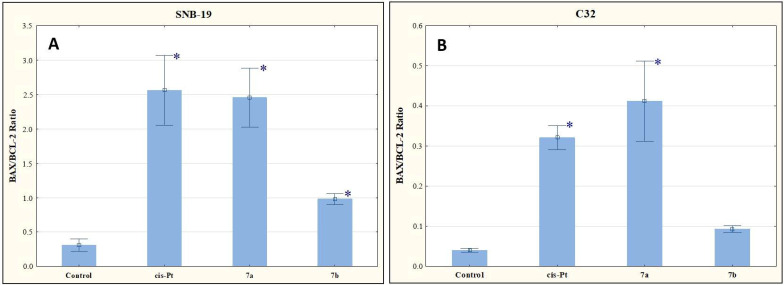
The effect of compounds **7a** and **7b** on BAX/BCL-2 for SNB-19 (**A**) and C-32 (**B**) cells. Cells were exposed to compound **7a** and **7b** for 24 h at a concentration equal to half of IC_50_ determined for SNB-19 (0.3 and 0.25 µg/mL, respectively) and for C-32 (0.7 and 0.25 µg/mL, respectively). For the reference compound, cisplatin, the appropriate concentrations were 0.8 µg/mL (SNB-19 line) and 0.6 µg/mL (C32 line). * *p* < 0.05 compared with the untreated control.

As shown in Figure 8, the tested compounds increased the mRNA copy number to BAX and BCL-2 ratio. Given this result, it can be assumed that the derivatives **7a** and **7b** affect apoptosis via the mitochondrial route.

### 2.4. Physicochemical Descriptors (Lipophilicity, ADME Properties)

For new derivatives, log P_TLC_ parameters were determined, and correlations between the values of lipophilicity parameters determined experimentally and calculated with the use of computer programs were ascertained.

Lipophilicity is an important physicochemical parameter related to the biological activity of compounds. This descriptor, contained in Lipiński’s rule of five, concerns the transport of a compound through the biological membrane system and the formation of a compound–receptor complex [39]. To assess the lipophilic nature of triterpenoids **3**–**5**, **6a**–**6e**, and **7a**–**7e**, the reverse phase chromatography (RP-TLC) method was used (chromatographic procedure by RP-TLC is included in the Supplementary Materials). The theoretical characteristics of the chromatographic parameters (R_M0_, log P_TLC_) of the tested derivatives were also performed. The results are presented in Table 6.

The lowest value of experimental lipophilicity (log P_TLC_ = 4.914) was shown for derivative **5** with two hydroxyl groups in positions C3 and C28. The conversion of these substituents into ester functions increased the lipophilicity of derivatives **6a**–**6e** and **7a**–**7e**. The highest increase in the log P_TLC_ value in the group of tested triterpenoids was recorded for the **6d** diester, which is related to the presence of two phenylpropynoyl groups.

The **7a**–**7e** monoesters are characterized by lower log P_TLC_ values than the compounds **6a**–**6e** containing two ester groups. Depending on the structure of the substituent, the monoesters can be ranked in the following order of increasing lipophilicity propargyloxycarbonyl < propynoyl < 2-butynoyl < cyclopropylpropynoyl < phenylpropynoyl. The derivatives **7a** and **7e** with a terminal triple bond possess the lowest lipophilicity (log P_TLC_- 6.937 and 6.916, respectively) compared to the other alkynyl-substituted triterpenoids.

The experimentally determined lipophilicity of the tested compounds was compared with the theoretical values. Additionally, the partition coefficients (log P) were calculated by use of the seven theoretical programs such as ALOGPs, AC logP, miLogP, ALOGP, MLOGP, XLOGP2, and XLOGP3 [40]. The results (Table S5, Supplementary Materials) show that compound **5** has the lowest lipophilicity, which is consistent with its log P_TLC_ value. In the case of the most lipophilic compound **6d**, the same relationship was found.

The calculated theoretical values of log P of compounds **3**–**5**, **6a**–**6e**, and **7a**–**7e** are in the range of 5.15–13.40. The highest log P values were obtained with the XLOGP3 and the lowest with the ALOGPs.

The correlation matrix for theoretical lipophilicity parameters of derivatives **3**–**5**, **6a**–**6e**, and **7a**–**7e** are presented in Table S6 (Supplementary Materials). The calculated correlation coefficients are in the range 0.812–0.987, and the highest value of correlation is found in the cases of XLOGP2 and XLOGP3 (0.987). These programs calculate the value of the log P parameter using the same method based on the additive atom/group model.

**Figure 9:**
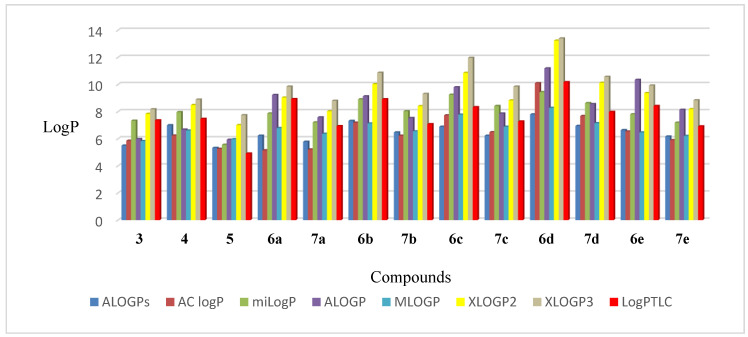
The values of lipophilicity of triterpenoids **3**–**5**, **6a**–**6e**, and **7a**–**7e**.

The comparison of the experimental lipophilicity values (log P_TLC_) for the derivatives **3**–**5**, **6a**–**6e**, and **7a**–**7e**, with the corresponding values obtained by different calculation methods, is depicted in Figure 9. The highest correlation between theoretically and experimentally received lipophilicity values were obtained in the case of the XLOGP2 program (0.861), while the lowest was in the case of MLOGP (0.763).

The Log P_TLC_ values of the most active compounds **7a** and **7b** range from 6.937 to 7.982. This experimental lipophilicity does not comply with Lipiński’s rule (log P < 5). This means that lipophilicity is not the only possible factor influencing the biological activity of a compound.

For active monoesters **7a**–**7e**, good correlation between antiproliferative activity against the C-32 and SNB-19 cell lines and lipophilicity could be observed. The obtained relationships described by the equations (A and B) are presented in Table 7.

In silico pharmacokinetic analysis is an important method for verifying and simulating the potential biological activity of a future drug substance. The main goal of early-phase pharmacokinetic studies is the preselection of newly synthesized chemical compounds and qualifying structures with optimal properties for further study. The bioavailability studies of compounds, in addition to the lipophilicity parameter, also include the determination of such descriptors as molecular weight (M), topological polar surface of the molecule (tPSA), log BB (Brain/blood partition coefficient), number of rotatable bonds (nROTB), and number of donor (nHBD) and acceptor (nHBA) sites for hydrogen bonds [41]. All ADME parameters for derivatives **3**–**5**, **6a**–**6e**, and **7a**–**7e** were calculated using ACD/iLab software and tabulated in Table 8 [42].

The values of nHBA (≤10), nHBD (≤5), and tPSA (<140 Å^2^) determined the oral bioavailability of the 30-diethylphosphate derivatives of betulin.

Essentially, the determination of the brain/blood barrier (BBB) permeability parameter has two aspects. The first is to identify neurotoxic substances that damage the brain. In addition, it may be helpful in designing new drugs that can achieve the goal in the CNS [43]. The presence of BBB makes the treatment of glioblastoma very difficult, as the chemotherapeutic agents used cannot be delivered to the target tumor cells. The triterpenes **3**–**5**, **6a**–**6e**, and **7a**–**7e** have the log BB values that fit within an optimal range of the values for drug-likeness (log BB −3 to 1.2) [44]. On the other hand, the log BB values obtained for the tested phosphate derivatives (active in vitro against SNB-19 cells) are characteristic of drugs that are inactive in the CNS. However, only a small number of low molecular weight fat-soluble compounds have the ability to pass through the BBB. Other molecules need a special transport system for this. The use of such forms as liposomes, dendrimers, or polymeric micelles allows chemotherapeutic agents to overcome this barrier and reach the goal of action [45].

It is also known that in the cancer process, some primary tumor cancer cells may metastasize. Such cells have the ability to migrate and can colonize even distant tissues [46]. Metastasis is a significant problem in the treatment of neoplastic diseases and contributes to the worsening of the patient’s prognosis. Metastasis of glioblastoma beyond the nervous system is determined at the level of about 2% of cases, but the number of this type of metastasis is increasing. This is due to the greater effectiveness of the primary tumor treatment, which extends the survival time. Glioma metastases are most often diagnosed in the lungs and pleura (60%) [47].

### 2.5. In Silico Study

As already mentioned, abnormalities in the expression of genes regulating the cell cycle play an important role in the process of cancer development. Both the initiation and development of the neoplastic process are related not only to such factors as mutations of proto-oncogenes and suppressor genes, disturbances in the DNA repair process, and mutations of anti- and pro-apoptotic genes. Increased expression of growth factors and angiogenesis also plays an important role in these processes. The epidermal growth factor receptor (EGFR) has been observed to be elevated in stomach, brain, and breast tumors. This receptor belongs to the receptor tyrosine kinase (RTK), the pathway of which is the most disrupted signaling cascade in glioblastoma [48,49].

**Figure 10:**
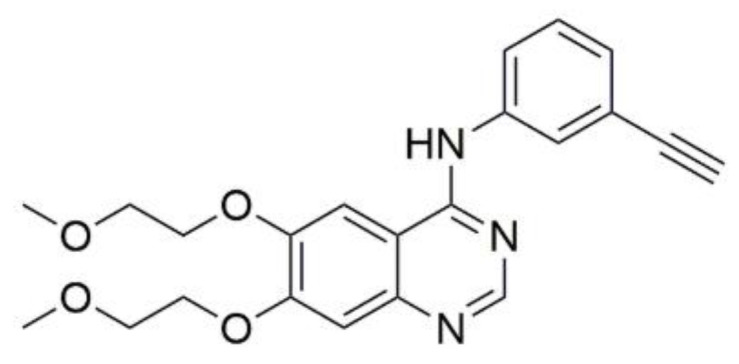
Structure of reference drug erlotinib.

According to our previous article on 3-substituted derivatives of betulin as potential EGFR inhibitors [19], molecular modeling was carried out to predict the possible mode of action of the newly synthesized compounds **7a** and **7b**. EGFR structure was obtained from PDB (PDB ID: 1M17). The co-crystallized ligand for 1M17 was a known EGFR inhibitor (i.e., erlotinib) and was taken as the reference drug (Figure 10).

**Figure 11:**
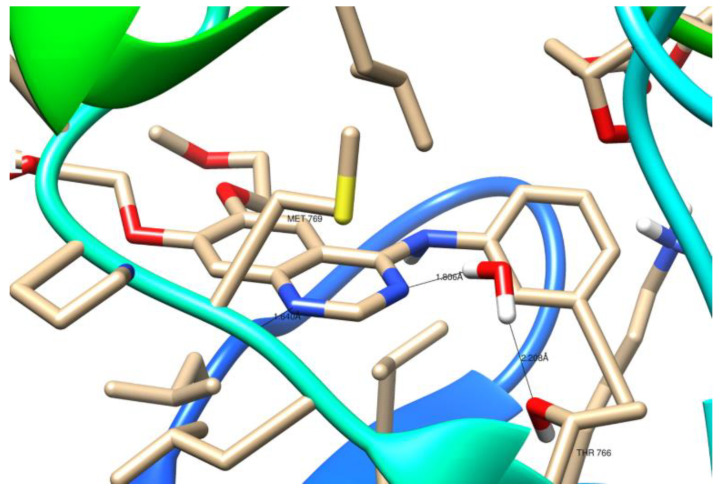
Three-dimensional (3D) binding interaction of erlotinib with EGFR (PDB ID: 1M17).

As illustrated in Figure 11, the crystal structure of 1M17 shows that N1 of the erlotinib quinazoline moiety receives a hydrogen bond from the amide nitrogen of Met769. Moreover, the water molecule (W10) essentially forms a hydrogen-bonding network, which involves nitrogen N3 of the erlotinib quinazoline moiety and the Thr766 side chain.

Genetic Optimization for Ligand Docking (GOLD) ver. 2020.1 [50] was used for the hydrated docking analysis. GOLD is an automated ligand docking program that relies on the use of a genetic algorithm to study the full range of conformational flexibility of ligands with partial protein flexibility (flexibility of receptor hydrogens) [51]. The region of interest used for GOLD docking was defined as all the EGFR protein residues within the 10 Å of the reference ligand. For the remaining water molecules, the “Toggle” option was on (having GOLD decide whether the water should be present or absent, i.e., bound or displaced by the ligand during docking). The results of docking compounds **7a** and **7b** to the active site of EGFR tyrosine kinase obtained in the GOLD program are presented in Table 9. Ligand matches are presented in the arbitrary units [a.u.] of GOLD computer program. The higher the calculated value, the better the binding affinities of the ligand for its protein. The obtained results show that compound **7a** has a slightly lower docking score compared to the reference erlotinib (30.87 a.u. and 31.06 a.u., respectively). In contrast, compound **7b** shows a better binding affinity for EGFR compared to erlotinib (docking score 31.33 a.u.).

Binding energy is an important parameter for assessing the stability of the ligand-protein complex. It is released when a ligand molecule binds to a protein, which lowers the total energy of the complex. Thus, the greater the energy released when binding a ligand to a protein, the greater the tendency of the ligand to associate with that protein. For this reason, a binding energy calculation has been made using the K_DEEP_ predictor. K_DEEP_ calculates binding energy ΔG [kcal/mol] using deep convolutional neural networks (DCNNs) model [52].

The K_DEEP_ calculations for the crystal structure of the complex of erlotinib with EGFR (1M17) and the complex obtained by docking erlotinib to EGFR tyrosine kinase domain showed that both of these complexes achieved significantly higher binding energy values compared to compounds **7a** and **7b** (Table 9). According to the obtained in K_DEEP_ results, compounds **7a**,**b** show a better binding affinity to the active site of EGFR compared to erlotinib.

**Figure 12:**
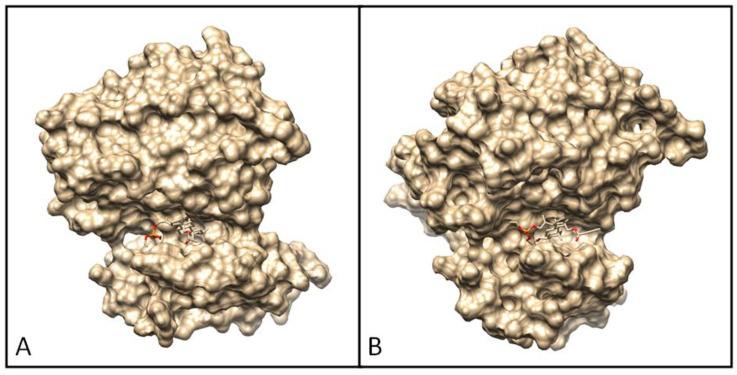
Docking pose of EGFR tyrosine kinase complex with **7a** (**A**) and **7b** (**B**).

**Figure 13:**
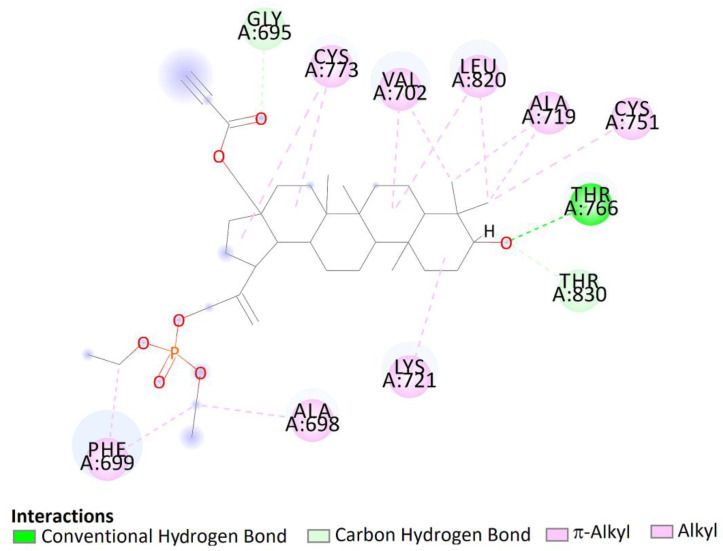
Two-dimensional (2D) binding interaction of **7a** with EGFR.

Docked poses of ligand **7a** and their interactions with amino acid residues are presented in Figure 12 and Figure 13.

The top docked pose of compound **7a** in the binding site showed hydrophobic interactions with Gly690, Thr830 (carbon hydrogen bond), Phe699 (π–alkyl), Ala698, Lys721, Cys751, Ala719, Leu820, Val702, and Cys773 (alkyl–alkyl). Moreover, hydroxy groups of triterpene in positions C3 were involved in hydrogen bonds to carboxyl oxygen atom of Thr766 (Figure 13).

**Figure 14:**
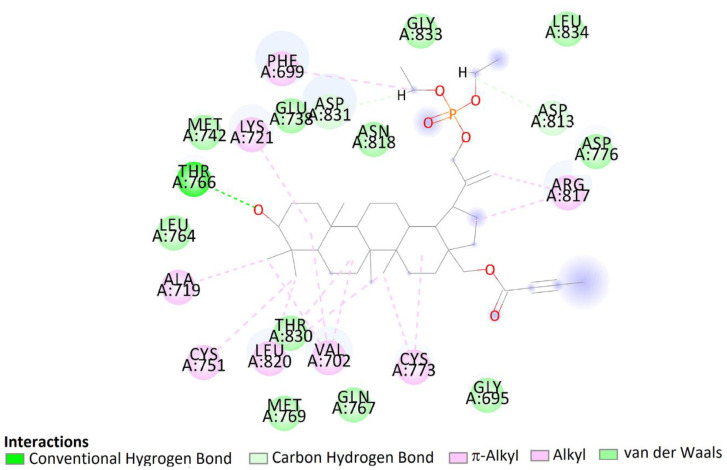
Two-dimensional (2D) binding interaction of **7b** with EGFR.

The pose of compound **7b** in the binding site showed hydrophobic interactions with 21 amino acids residues including interactions with Asp831, Phe832, and Gly833 (DFG motif). According to the obtained literature data, interactions with DFG motif play a significant role in tyrosine kinase activity [53]. Moreover, hydroxy groups of triterpene in positions C3 were involved in hydrogen bonds to carboxyl oxygen atom of Thr766, stabilizing the obtained complex (Figure 14).

Molecular dynamics (MD) calculation provide a better understanding of structure–function relationships in motion and other conformational changes by the proteins. Therefore, an MD study was conducted in this paper to evaluate the dynamic behavior of the top-scoring complexes for a period of 10 ns. The molecular mechanics/generalized born surface area (MM/GBSA) method was selected for rescoring complexes, because it is the fastest force-field based method that computes the free energy of binding, as compared to the other computational free energy methods, such as free energy perturbation (FEP) or thermodynamic integration (TI) methods [54]. Comparison studies have also shown that MM/GBSA outperforms molecular mechanics/Poisson Boltzmann surface area MM/PBSA [55]. The MM/GBSA calculation was performed using MolAICal software [56]. MolAICal provides a way to calculate the MM/GBSA based on the output results of molecular dynamics simulations that are carried out by NAMD [57]. The calculated binding free energies are shown in Table 10.

The binding free energies of **7a** and **7b** range from −46.53 to −43.51 kcal/mol, which are much smaller than those of erlotinib. According to the calculations performed, betulin derivatives **7a** and **7b** are better binders than the reference compound.

**Figure 15:**
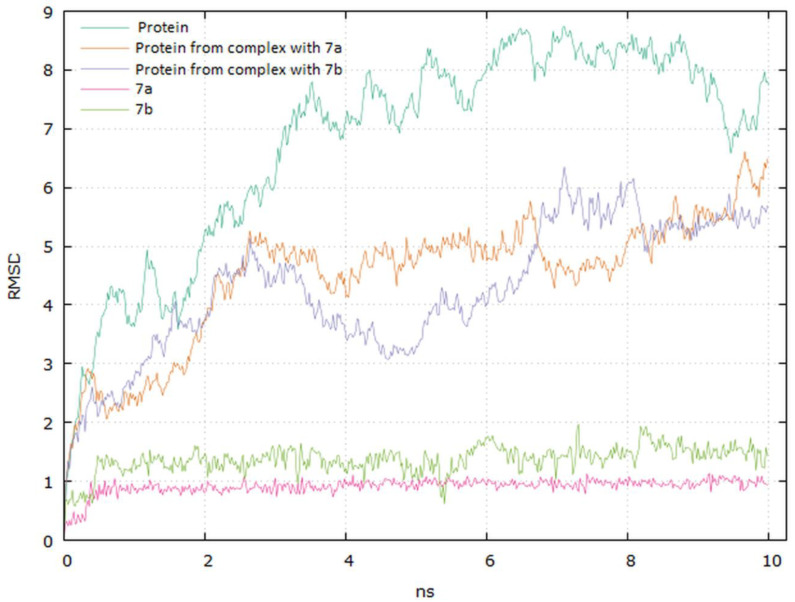
Graphical representation of the plots showing proteins CαRMSD (Å) and ligands atoms versus time (10 ns).

**Figure 16:**
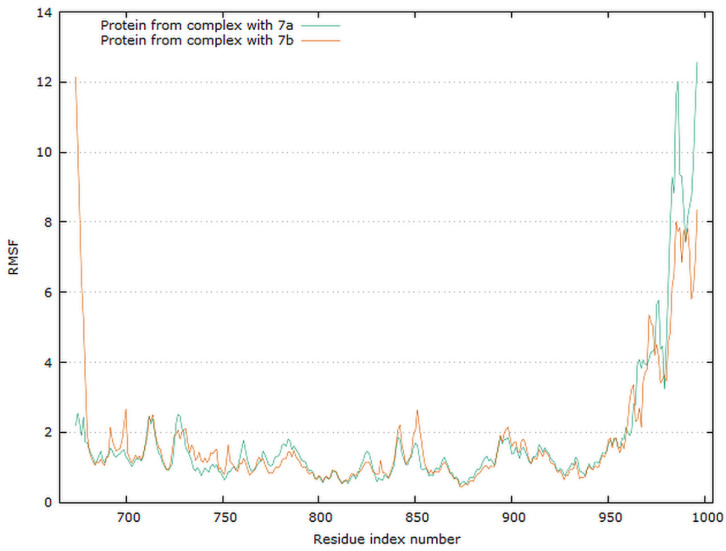
Graphical representation of the plots showing proteins RMSF [Å] versus residue index number of protein for **7a** and **7b** complexes of EGFR tyrosine kinase.

Analyzing the complexes ligand root-mean-square deviation (RMSD) and hydrogen bonding can provide insights into a structural conformation faced by the protein and ligand during simulation (Figure 15 and Figure 16).

The plots for EGFR proteins Cα versus time for the simulation with compounds **7a** and **7b** are shown in Figure 15. The visual analysis of the trajectory confirms the stability of the structure of protein at around 6 ns. It should be mentioned that the RMSD values obtained for the complexes were lower compared to the apo protein.

The plot for ligands RMSD versus time shows a plateau value of 1 Å and 1.3 Å for the ligands **7a** and **7b**, respectively, at around 0.3 ns. There was no a significant shift in RMSD values for the ligands backbone. The deviation value observed was within limits, which is quite stable for binding of the ligands with the EGFR tyrosine kinase. The higher value of RMSD for the **7b** compound may be due to the butynyl group at carboxylate moiety.

The residue root mean square fluctuation (RMSF) is a measure of the flexibility of a residue. It is typically calculated for the C_α_ atom of each residue. The plot for protein RMSF versus residue number index shown in Figure 16 describes fluctuations in the range of 0.5–2.5 Å for complexes EGFR with **7a** and **7b**. The fluctuations of the C-terminals ends were higher. The catalytic residues are no longer higher than 0.5 Å for both complexes, and are suitable for stable binding.

**Figure 17:**
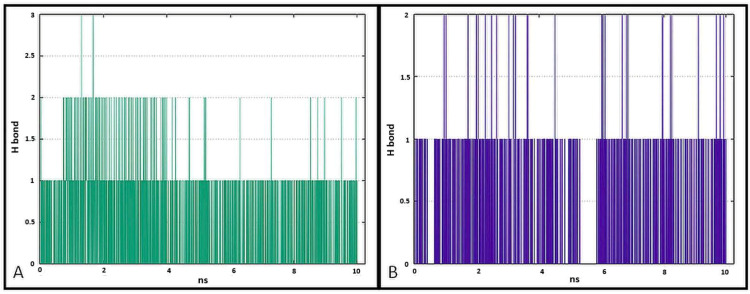
Pictorial representation of the number of H-bond contacts formed by ligand **7a** (**A**) and **7b** (**B**) with EGFR tyrosine kinase.

The data presented in Figure 17 show the results of analysis of hydrogen bond contacts in the studied complexes. Compound **7a** (Figure 17A) forms three hydrogen bonds, and the binding with Thr766 residue occurs in the complex at the 28% time of measurement. Ligand **7b** forms two hydrogen bonds during simulation time, including a stable H-bond with Thr766 (Figure 17B).

From the discussion, it can be summarized that the results obtained in silico are consistent with the experimental data, and compounds **7a** and **7b** have an efficient binding inside the cavity of EGFR tyrosine kinase.

## 3. Materials and Methods

### 3.1. General Experimental Procedures

Materials and methods are included in the Supplementary Materials.

3,28-Diacetyl-30-hydroxybetulin **3** was obtained in 52% yield by oxidation of 3,28-diacetylbetulin **2** with *m*-chloroperbenzoic acid in chloroform. Melting point and ^1^H and ^13^C NMR spectra for compound **3** were consistent with the literature data [58].

Phosphate derivatives of betulinic **8** and betulonic **9** acid were synthesized according to the described method. Spectroscopic (^1^H, ^13^C, and ^31^P NMR) and melting point data were consistent with the literature values [9].

### 3.2. Procedures for Synthesizing Compounds

#### 3.2.1. Synthesis of 3,28-Diacetoxy-30-diethoxyphosphoryloxybetulin **4**

A flask with a solution of compound **3** (0.54 g, 1 mmol) and 12 mg (0.1 mmol) 4-dimethylaminopyridine (DMAP) in 6 mL pyridine was placed in an ice-water bath. Then, diethyl chlorophosphate (0.28 mL, 1.9 mmol) was added dropwise to the cooled mixture, and stirring was continued for 24 h under an inert gas (argon) atmosphere after the cooling bath was removed. The course of the reaction was monitored using thin layer chromatography (TLC). After completion of the reaction, the pyridine was evaporated under reduced pressure. Dichloromethane (30 mL) was added to the residue, and the resulting solution was washed with 10% HCl (2 × 10 mL), saturated NaHCO_3_ (12 mL), and then with water (2 × 10 mL). The organic phase was dried with anhydrous Na_2_SO_4_ and then concentrated. The product was isolated by column chromatography (SiO_2_, hexane:ethyl acetate, 3:2, *v*/*v*).

Yield: 72%, m. p. 61–63 °C.

TLC (dichloromethane:ethanol, 15:1, *v*/*v*): R_f_ = 0.48. ^1^H-NMR (600 MHz, CDCl_3_) δ (ppm): 5.06 (s, 1H, H-29), 5.00 (s, 1H, H-29), 4.51 (m, 2H, H-30), 4.48 (m, 1H, H-3), 4.26 (d, *J* = 10.8 Hz, 1H, H-28), 4.16 (m, 4H, 2× OC*H*_2_CH_3_), 3.84 (d, *J* = 10.8 Hz, 1H, H-28), 2.39 (m, 1H, H-19), 2.06 (s, 3H, C(O)CH_3_), 2.09 (s, 3H, C(O)CH_3_), 0.85–2.00 (m, CH, CH_2_), 1.38 (m, 6H, 2× OCH_2_C*H*_3_), 1.08 (s, 3H, CH_3_), 1.01 (s, 3H, CH_3_), 0.87 (s, 3H, CH_3_), 0.86 (s, 3H, CH_3_), 0.85 (s, 3H, CH_3_), 0.80 (m, 1H, H-5). ^13^C-NMR (150 MHz, CDCl_3_) δ (ppm): 171.6; 171.0; 149.0; 110.5; 80.9; 63.8; 63.8; 62.4; 55.4; 50.2; 49.5; 46.3; 43.2; 42.7; 40.9; 38.4; 37.8; 37.5; 37.1; 34.3; 34.1; 31.3; 29.7; 28.0; 27.0; 26.6; 23.7; 21.3; 21.0; 20.9; 18.2; 16.2; 16.2; 16.1; 16.0; 14.9. ^31^P-NMR (243 MHz, CDCl_3_) δ (ppm): −0.91. HR-MS (APCI) *m*/*z*: C_38_H_62_O_8_P [(M–H)^−^], Calc. 677.4182; Found 677.4180.

#### 3.2.2. Synthesis of 30-Diethoxyphosphoryloxybetulin **5**

A solution of 730 mg sodium hydroxide in 10 mL of methanol, 20 mL of tetrahydrofuran, and 10 mL of water was prepared in the flask. Then, 680 mg of compound **4** (1 mmol) was added. The reaction was carried out at room temperature while stirring with a magnetic stirrer. The progress of the reaction was monitored by TLC (SiO_2_, dichloromethane:ethanol, 15:1, *v*/*v*). After 5 h, the reaction mixture was evaporated to dryness. The residue was dissolved in dichloromethane and washed with 10 mL of hydrochloric acid and then three times with 30 mL of water. The organic phase was dried with anhydrous Na_2_SO_4_, and the product was purified using column chromatography (SiO_2_, dichloromethane:ethanol, 15:1, *v*/*v*).

Yield: 85%, m. p. 194–195 °C.

TLC (dichloromethane:ethanol, 15:1, *v*/*v*): R_f_ = 0.36. ^1^H-NMR (600 MHz, CDCl_3_) δ (ppm): 5.49 (d, *J* = 1.2 Hz, 1H, H-29); 4.99 (s, 1H, H-29), 4.51 (m, 2H, H-30), 4.15 (m, 4H, 2× OC*H*_2_CH_3_), 3.80 (d, *J* = 10.8 Hz, 1H, H-28), 3.32 (d, *J* = 10.8 Hz, 1H, H-28), 3.21 (m, 1H, H-3), 2.35 (m, 1H, H-19), 0.90–2.00 (m, CH, CH_2_), 1.37 (m, 6H, 2× OCH_2_C*H*_3_), 1.07 (s, 3H, CH_3_), 1.04 (s, 3H, CH_3_), 1.00 (s, 3H, CH_3_), 0.84 (s, 3H, CH_3_), 0.78 (s, 3H, CH_3_), 0.70 (m, 1H, H-5). ^13^C-NMR (150 MHz, CDCl_3_) δ (ppm): 148.3; 109.2; 77.9; 68.0; 2 × 62.8; 59.2; 54.2; 49.3; 48.5; 46.7; 42.1; 41.6; 39.9; 37.8; 37.7; 36.2; 36.1; 33.2; 32.7; 30.5; 28.2; 27.0; 26.3; 26.0; 25.7; 19.9; 17.3; 2 × 15.2; 15.0; 14.3, 13.7. ^31^P-NMR (243 MHz, CDCl_3_) δ (ppm): −0.91. HR-MS (APCI) *m*/*z*: C_34_H_58_O_6_P [(M–H)^−^], Calc. 593.3971; Found 593.3959.

#### 3.2.3. General Procedure for Synthesis of Alkynyl Derivatives **6a**–**6d** and **7a**–**7d**

A solution of 595 mg of 30-diethoxyphosphoryloxybetulin **5** (1 mmol) in 5 mL of dichloromethane was cooled in an ice–salt bath to −10 °C, and 1.17 mmol of the corresponding alkynyl acid was added. Then, a solution of 240 mg of DCC and 10 mg of DMAP in 1 mL of dichloromethane was slowly added dropwise. The reaction was carried out under an argon atmosphere while stirring with a magnetic stirrer. The flask with the reaction mixture was kept in an ice–salt bath for the first 5 h, after which the reaction was carried out at room temperature. After 24 h, the precipitate was filtered off and the filtrate was concentrated to dryness in a vacuum evaporator. Subsequently, the reaction products were purified using column chromatography (SiO_2_, dichloromethane:ethanol, 15:1 or 40:1, *v*/*v*).

*30-Diethoxyphosphoryloxy-3,28-dipropynoylbetulin* **6a**

Yield: 5%, m. p. 127–130 °C.

TLC (dichloromethane:ethanol, 15:1, *v*/*v*): R_f_ = 0.75. ^1^H-NMR (600 MHz, CDCl_3_) δ (ppm): 5.07 (s, 1H, H-29), 5.00 (s, 1H, H-29), 4.62 (m, 1H, H-3), 4.51 (m, 2H, H-30), 4.40 (d, *J* = 10.8 Hz, 1H, H-28), 4.15 (m, 4H, 2× OC*H*_2_CH_3_), 3.98 (d, *J* = 10.8 Hz, 1H, H-28), 2.92 (s, 1H, C≡CH), 2.88 (s, 1H, C≡CH), 2.38 (m, 1H, H-19), 0.93–2.10 (m, CH, CH_2_), 1.37 (m, 6H, 2× OCH_2_C*H*_3_), 1.05 (s, 3H, CH_3_), 1.00 (s, 3H, CH_3_), 0.91 (s, 3H, CH_3_), 0.90 (s, 3H, CH_3_), 0.88 (s, 3H, CH_3_), 0.74 (m, 1H, H-5). ^13^C-NMR (150 MHz, CDCl_3_) δ (ppm): 153.2; 152.8; 148.8; 110.7; 83.6; 75.0; 74.6; 2 × 68.9; 64.4; 63.9; 63.9; 55.3; 50.2; 49.5; 46.4; 43.1; 42.7; 40.9; 38.3; 37.9; 37.7; 37.5; 37.0; 34.2; 34.1; 31.2; 29.6; 27.9; 26.9; 26.6; 23.5; 20.9; 18.1; 16.5; 2 × 16.2; 16.1; 14.7. ^31^P-NMR (243 MHz, CDCl_3_) δ (ppm): −0.93. HR-MS (APCI) *m*/*z*: C_40_H_58_O_8_P [(M–H)^−^], Calc. 697.3869; Found 697.3856.

*3,28-Di(2-butynoyl)-30-diethoxyphosphoryloxybetulin* **6b**

Yield: 10%, m. p. 66–70 °C.

TLC (dichloromethane:ethanol, 40:1, *v*/*v*): R_f_ = 0.73. ^1^H-NMR (600 MHz, CDCl_3_) δ (ppm): 5.06 (s, 1H, H-29), 4.99 (s, 1H, H-29), 4.59 (m, 1H, H-3), 4.50 (m, 2H, H-30), 4.34 (d, *J* = 10.8 Hz, 1H, H-28), 4.16 (m, 4H, 2× OC*H*_2_CH3), 3.93 (d, *J* = 10.8 Hz, 1H, H-28), 2.37 (m, 1H, H-19), 2.02 (s, 3H, CH_3_C≡C), 2.01 (s, 3H, C≡CCH_3_), 0.93–2.10 (m, CH, CH_2_), 1.36 (m, 6H, 2× OCH_2_C*H*_3_), 1.04 (s, 3H, CH_3_), 0.99 (s, 3H, CH_3_), 0.90 (s, 3H, CH_3_), 0.89 (s, 3H, CH_3_), 0.86 (s, 3H, CH_3_), 0.80 (m, 1H, H-5). ^13^C-NMR (150 MHz, CDCl_3_) δ (ppm): 154.3; 153.9; 149.1; 111.4; 85.7; 85.0; 82.8; 72.5; 72.5; 69.0; 63.9; 63.8; 55.4; 50.2; 46.4; 45.5; 42.7; 40.9; 38.4; 37.9; 37.5; 37.0; 34.2; 34.1; 30.6; 29.6; 27.9; 27.3; 27.0; 23.6; 20.9; 19.7; 18.1; 16.5; 2 × 16.2; 16.1; 16.0; 14.7; 3.9; 3.9. ^31^P-NMR (243 MHz, CDCl_3_) δ (ppm): −0.90. HR-MS (APCI) *m*/*z*: C_42_H_62_O_8_P [(M-H)^−^], Calc. 725.4182; Found 725.4162.

*3,28-Di(2-cyclopropylpropynoyl)-30-diethoxyphosphoryloxybetulin* **6c**

Yield: 8%, m. p. 67–70 °C.

TLC (dichloromethane:ethanol, 15:1, *v*/*v*): R_f_ = 0.70. ^1^H-NMR (600 MHz, CDCl_3_) δ (ppm): 5.05 (s, 1H, H-29), 4.98 (s, 1H, H-29), 4.58 (m, 1H, H-3), 4.50 (m, 2H, H-30), 4.31 (d, *J* = 10.8 Hz, 1H, H-28), 4.14 (m, 4H, 2× OC*H*_2_CH_3_), 3.91 (d, *J* = 10.8 Hz, 1H, H-28), 2.37 (m, 1H, H-19), 0.93–2.10 (m, CH, CH_2_), 1.36 (m, 6H, 2× OCH_2_C*H*_3_), 1.02 (s, 3H, CH_3_), 0.97 (s, 3H, CH_3_), 0.95 (m, 2 × 2H, CH_2_C≡CH), 0.93 (m, 2 × 2H, CH_2_C≡CH), 0.88 (s, 3H, CH_3_), 0.87 (s, 3H, CH_3_), 0.85 (s, 3H, CH_3_), 0.77 (m, 1H, H-5). ^13^C-NMR (150 MHz, CDCl_3_) δ (ppm): 154.9; 154.5; 149.4; 111.1; 94.0; 93.3; 83.1; 69.4; 2 × 69.0; 64.4; 2 × 64.2; 55.9; 50.7; 50.0; 47.0; 43.2; 41.4; 38.5; 38.3; 38.0; 37.5; 34.8; 34.6; 30.1; 28.4; 27.5; 27.1; 24.1; 21.4; 18.7; 17.1; 16.7; 16.7; 16.5; 15.2; 2 × 9.7; 0.0. ^31^P-NMR (243 MHz, CDCl_3_) δ (ppm): −0.91. HR-MS (APCI) *m*/*z*: C_37_H_58_O_6_P [(M–H)^−^], Calc. 629.3971; Found 629.3965.

*30-Diethoxyphosphoryloxy-3,28-di(2-phenylpropynoyl)betulin* **6d**

Yield: 12%, m. p. 89–91 °C.

TLC (dichloromethane:ethanol, 40:1, *v*/*v*): R_f_ = 0.77. ^1^H-NMR (600 MHz, CDCl_3_) δ (ppm): 7.62 (m, 4H, H_arom_), 7.47 (m, 2H, H_arom_), 7.40 (m, 4H, H_arom_), 5.07 (s, 1H, H-29), 5.01 (s, 1H, H-29), 4.68 (m, 1H, H-3), 4.52 (m, 2H, H-30), 4.43 (d, *J* = 10.8 Hz, 1H, H-28), 4.17 (m, 4H, 2× OC*H*_2_CH_3_), 4.02 (d, *J* = 10.8Hz, 1H, H-28), 2.41 (m, 1H, H-19), 0.93–2.10 (m, CH, CH_2_), 1.38 (m, 6H, 2× OCH_2_C*H*_3_), 1.08 (s, 3H, CH_3_), 1.02 (s, 3H, CH_3_), 0.95 (s, 3H, CH_3_), 0.94 (s, 3H, CH_3_), 0.90 (s, 3H, CH_3_), 0.84 (m, 1H, H-5). ^13^C-NMR (150 MHz, CDCl_3_) δ (ppm): 154.7; 154.2; 149.0; 2 × 133.0; 2 × 130.5; 2 × 129.7; 2 × 128.5; 2 × 119.9; 110.7; 86.5; 85.8; 83.2; 81.1; 80.7; 68.9; 64.1; 2 × 63.8; 55.4; 50.2; 46.5; 42.7; 40.9; 38.4; 38.0; 37.6; 37.1; 34.3; 34.1; 31.2; 29.7; 28.0; 17.3; 27.0; 26.6; 23.6; 20.9; 18.2; 16.6; 2 × 16.2; 16.1; 14.8. ^31^P-NMR (243 MHz, CDCl_3_) δ (ppm): −0.88. HR-MS (APCI) *m*/*z*: C_37_H_58_O_6_P [(M–H)^−^], Calc. 849.4495; Found 849.4483.

*30-Diethoxyphosphoryloxy-28-propynoylbetulin* **7a**

Yield: 72%, m. p. 177–180 °C.

TLC (dichloromethane:ethanol, 15:1, *v*/*v*): R_f_ = 0.51. ^1^H-NMR (600 MHz, CDCl_3_) δ (ppm): 5.07 (s, 1H, H-29), 5.00 (s, 1H, H-29), 4.51 (m, 2H, H-30), 4.40 (d, *J* = 10.8Hz, 1H, H-28), 4.15 (m, 4H, 2× OC*H*_2_CH_3_), 3.99 (d, *J* = 10.8 Hz, 1H, H-28), 3.20 (m, 1H, H-3), 2.92 (s, 1H, C≡CH), 2.38 (m, 1H, H-19), 0.93–2.10 (m, CH, CH_2_), 1.37 (m, 6H, 2× OCH_2_C*H*_3_), 1.04 (s, 3H, CH3), 1.01 (s, 3H, CH_3_), 0.99 (s, 3H, CH_3_), 0.84 (s, 3H, CH_3_), 0.79 (s, 3H, CH_3_), 0.69 (m, 1H, H-5). ^13^C-NMR (150 MHz, CDCl_3_) δ (ppm): 153.2; 148.8; 110.7; 78.9; 74.8; 74.7; 68.9; 64.5; 2 × 63.9; 55.3; 50.3; 49.5; 46.4; 43.1; 42.7; 40.9; 38.9; 38.7; 37.6; 37.1; 34.2; 33.6; 31.2; 29.6; 28.0; 27.4; 27.0; 26.7; 20.8; 18.4; 18.3; 2 × 16.2; 16.0; 15.4; 14.8. ^31^P-NMR (243 MHz, CDCl_3_) δ (ppm): −0.94. HR-MS (APCI) *m*/*z*: C_37_H_58_O_7_P [(M–H)^−^], Calc. 645.3920; Found 645.3918.

*28-(2-Butynoyl)-30-diethoxyphosphoryloxybetulin* **7b**

Yield: 81%, m. p. 133–136 °C.

TLC (dichloromethane:ethanol, 15:1, *v*/*v*): R_f_ = 0.52. ^1^H-NMR (600 MHz, CDCl_3_) δ (ppm): 5.06 (s, 1H, H-29), 4.99 (s, 1H, H-29), 4.50 (m, 2H, H-30), 4.35 (d, *J* = 10.8Hz, 1H, H-28), 4.15 (m, 4H, 2× OC*H*_2_CH_3_), 3.94 (d, *J* = 10.8 Hz, 1H, H-28), 3.20 (m, 1H, H-3), 2.38 (m, 1H, H-19), 2.01 (s, 3H, C≡CCH_3_), 0.93–2.10 (m, CH, CH_2_), 1.37 (m, 6H, 2× OCH_2_C*H*_3_), 1.04 (s, 3H, CH_3_), 0.99 (s, 3H, CH_3_), 0.98 (s, 3H, CH_3_), 0.83 (s, 3H, CH_3_), 0.78 (s, 3H, CH_3_), 0.70 (m, 1H, H-5). ^13^C-NMR (150 MHz, CDCl_3_) δ (ppm): 154.3; 148.9; 110.5; 85.5; 78.9; 72.5; 68.8; 63.8; 63.8; 55.3; 50.3; 49.5; 46.4; 43.1; 42.7; 40.9; 38.9; 38.7; 37.5; 37.1; 34.2; 31.2; 29.6; 28.0; 27.4; 27.0; 26.6; 20.8; 18.3; 2 × 16.2; 16.1; 15.4; 14.8, 3.9. ^31^P-NMR (243 MHz, CDCl_3_) δ (ppm): −0.91. HR-MS (APCI) *m*/*z*: C_38_H_60_O_7_P [(M–H)-], Calc. 659.4077; Found 659.4079.

*28-(2-Cyclopropylpropynoyl)-30-diethoxyphosphoryloxybetulin* **7c**

Yield: 82%, m. p. 133–134 °C.

TLC (dichloromethane:ethanol, 15:1, *v*/*v*): R_f_ = 0.54. ^1^H-NMR (600 MHz, CDCl_3_) δ (ppm): 5.05 (s, 1H, H-29), 4.95 (s, 1H, H-29), 4.50 (m, 2H, H-30), 4.32 (d, *J* = 10.8Hz, 1H, H-28), 4.15 (m, 4H, 2× OC*H*_2_CH_3_), 3.92 (d, *J* = 10.8 Hz, 1H, H-28), 3.20 (m, 1H, H-3), 2.38 (m, 1H, H-19), 0.93–2.10 (m, CH, CH_2_), 1.36 (m, 6H, 2× OCH_2_C*H*_3_), 1.03 (s, 3H, CH_3_), 0.99 (s, 3H, CH_3_), 0.98 (s, 3H, CH_3_), 0.95 (s, 2H, C≡CCHC*H*_2_), 0.93 (s, 2H, C≡CCHC*H*_2_), 0.83 (s, 3H, CH_3_), 0.77 (s, 3H, CH_3_), 0.69 (m, 1H, H-5). ^13^C-NMR (150 MHz, CDCl_3_) δ (ppm): 155.0; 149.4; 111.0; 94.0; 79.5; 69.3; 69.0; 2 × 64.4; 64.2; 55.8; 50.8; 50.0; 43.5; 43.2; 41.4; 39.4; 39.2; 39.0; 38.1; 37.7; 34.8; 34.7; 34.4; 31.7; 30.2; 28.5; 27.9; 27.5; 26.0; 21.4; 18.8; 2 × 16.7; 16.6; 15.9; 15.3; 9.75. ^31^P-NMR (243 MHz, CDCl_3_) δ (ppm): −0.93. HR-MS (APCI) *m*/*z*: C_40_H_62_O_7_P [(M–H)^−^], Calc. 685.4233; Found 685.4229.

*30-Diethoxyphosphoryloxy-28-(2-phenylpropynoyl)betulin* **7d**

Yield: 71%, m. p. 131–134 °C.

TLC (dichloromethane:ethanol, 40:1, *v*/*v*): R_f_ = 0.52. ^1^H-NMR (600 MHz, CDCl_3_) δ (ppm): 7.62 (m, 2H, H_arom_), 7.47 (m, 1H, H_arom_), 7.40 (m, 2H, H_arom_), 5.07 (s, 1H, H-29), 5.01 (s, 1H, H-29), 4.51 (m, 2H, H-30), 4.42 (d, *J* = 10.8 Hz, 1H, H-28), 4.16 (m, 4H, 2× OC*H*_2_CH_3_), 4.03 (d, *J* = 10.8Hz, 1H, H-28), 3.20 (m, 1H, H-3), 2.41 (m, 1H, H-19), 0.93–2.10 (m, CH, CH_2_), 1.37 (m, 6H, 2× OCH_2_C*H*_3_), 1.06 (s, 3H, CH_3_), 1.01 (s, 3H, CH_3_), 0.99 (s, 3H, CH_3_), 0.84 (s, 3H, CH_3_), 0.78 (s, 3H, CH_3_), 0.69 (m, 1H, H-5). ^13^C-NMR (150 MHz, CDCl_3_) δ (ppm): 154.6; 148.9; 133, 130.6; 129.7; 128.6; 119.7; 110.6; 86.4; 80.7; 78.9; 68.8; 64.1; 2 × 63.8; 55.3; 50.3; 49.5; 46.5; 43.2; 42.7; 40.9; 38.9; 38.2; 37.6; 37.1; 34.3; 34.2; 31.62; 29.7; 28.0; 27.4; 27.0; 26.7; 20.9; 19.7; 18.3; 2 × 16.2; 16.1; 15.4; 14.8. ^31^P-NMR (243 MHz, CDCl_3_) δ (ppm): −0.91. HR-MS (APCI) *m*/*z*: C_43_H_62_O_7_P [(M–H)^−^], Calc. 721.4233; Found 721.4226.

#### 3.2.4. Synthesis of Alkynyl Derivatives **6e** and **7e**

A solution of compound **5** (595 mg, 1mmol) in 4.5 mL of dry benzene and 3.75 mL of pyridine was cooled to about −5 °C. Then, 1.5 mmol of propargyl chloroformate in 4 mL of benzene was gradually added dropwise while stirring with a magnetic stirrer. Stirring was continued for 18 h at room temperature. The volatiles were evaporated, 6 mL of chloroform was added to the residue, and the resulting solution was washed with 10% sulfuric acid and then with water. After drying over anhydrous sodium sulfate, the chloroform was removed in a vacuum evaporator. The mono- and disubstituted derivatives, **6e** and **7e**, were isolated using column chromatography (SiO_2_, dichloromethane:ethanol 15: 1, *v*/*v*).

*30-Diethoxyphosphoryloxy-3,28-dipropargyloxycarbonyloxybetulin* **6e**

Yield: 15%, m. p. 129–131 °C.

TLC (dichloromethane:ethanol, 15:1, *v*/*v*): R_f_ = 0.82. ^1^H-NMR (600 MHz, CDCl_3_) δ (ppm): 4.98 (s, 1H, H-29), 4.91 (s, 1H, H-29), 4.67 (m, 2H, OCH_2_-C≡C), 4.65 (m, 2H, OCH_2_-C≡C), 4.29 (d, *J* = 6 Hz, 2H, H-30), 4.30 (d, *J* = 10.8 Hz, 1H, H-28), 4.27 (m, 1H, H-3), 4.07 (m, 4H, 2× OC*H*_2_CH_3_), 3.85 (d, *J* = 10.8 Hz, 1H, H-28), 2.47 (t, *J* = 2.4 Hz, 1H, C≡CH), 2.45 (t, *J* = 2.4 Hz, 1H, C≡CH), 2.28 (m, 1H, H-19), 1.0–2.10 (m, CH, CH_2_), 1.29 (m, 6H, 2× OCH_2_C*H*_3_), 0.96 (s, 3H, CH_3_), 0.91 (s, 3H, CH_3_), 0.85 (s, 3H, CH_3_), 0.78 (s, 3H, CH_3_), 0.77 (s, 3H, CH_3_), 0.70 (m, 1H, H-5). ^13^C-NMR (150 MHz, CDCl_3_) δ (ppm): 155.1; 154.6; 148.8; 110.7; 86.2; 75.7; 75.5; 68.9; 66.9; 63.9; 63.8; 2 × 55.3; 55.0; 53.5; 50.2; 49.5; 46.6; 42.7; 40.9; 38.3; 38.1; 37.5; 37.0; 34.1; 31.2; 29.5; 27.6; 26.9; 26.6; 23.6; 20.9; 18.1; 16.4; 2 × 16.2; 16.1; 16.0; 14.8. ^31^P-NMR (243 MHz, CDCl_3_) δ (ppm): −0.9. HR-MS (APCI) *m*/*z*: C_42_H_62_O_10_P [(M–H)^−^], Calc. 757.4081; Found 757.4075.

*30-Diethoxyphosphoryloxy-28-propargyloxycarbonyloxybetulin* **7e**

Yield: 60%, m. p. 172–174 °C.

TLC (dichloromethane:ethanol, 15:1, *v*/*v*): R_f_ = 0.64. ^1^H-NMR (600 MHz, CDCl_3_) δ (ppm): 4.97 (s, 1H, H-29), 4.91 (s, 1H, H-29), 4.67 (dd, *J* = 1.2 Hz, *J* = 2.4 Hz, 2H, OCH_2_-C≡C), 4.42 (d, *J* = 6 Hz, 2H, H-30), 4.30 (d, *J* = 10.8 Hz, 1H, H-28), 4.07 (m, 4H, 2× OC*H*_2_CH_3_), 3.85 (d, *J* = 10.8 Hz, 1H, H-28), 3.11 (m, 1H, H-3), 2.47 (t, *J* = 2.4 Hz, 1H, C≡CH), 2.47 (m, 1H, H-19), 0.93–2.10 (m, CH, CH_2_), 1.28 (m, 6H, 2× OCH_2_C*H*_3_), 0.96 (s, 3H, CH_3_), 0.91 (s, 3H, CH_3_), 0.90 (s, 3H, CH_3_), 0.75 (s, 3H, CH_3_), 0.69 (s, 3H, CH_3_), 0.61 (m, 1H, H-5). ^13^C-NMR (150 MHz, CDCl_3_) δ (ppm): 155.1; 148.9; 110.6; 78.9; 75.7; 68.9; 66.9; 63.9; 63.8; 55.3; 55.2; 50.3; 49.5; 46.6; 42.7; 40.9; 38.9; 38.7; 37.5; 37.1; 34.2; 34.1; 31.2; 29.5; 28.0; 27.4; 27.0; 26.7; 20.8; 18.3; 2 × 16.2; 16.1; 16.0; 15.4; 14.8. ^31^P-NMR (243 MHz, CDCl_3_) δ (ppm): −0.91. HR-MS (APCI) *m*/*z*: C_38_H_60_O_8_P [(M–H)^−^], Calc. 675.4026; Found 675.4019.

### 3.3. Raman Spectroscopy

Raman spectra were measured using the Raman confocal microscope Alfa 300R (WITec Instruments Corp., Germany) equipped with an Olympus 50×/0.50 long working distance objective and 532 nm wavelength diode laser. Averaged spectra were accumulated 100 times with a 2 s integration time, at the range 200–3800 cm^−1^ and with a spectral resolution about 1.5 cm^−1^ (at 2000 cm^−1^). The power of the laser beam on the sample did not exceed 10 mW, which prevented heating of the samples. OriginPro 9.1 software was used to analyze the spectra. The spectra were smoothed (Savitzky–Golay method, seven points) and background subtracted. The band positions were determined by the curve fitting (Gaussian function shape) with accuracy of about 1 cm^−1^.

The theoretical vibrational spectra of compounds **5** and **10** were calculated using Gaussin09 software. Structure optimization and spectra calculations were performed with DFT B3LYP hybrid functional and 6–31G(d) basis set. No imaginary frequencies were found for the optimized structures, which means that at least local energy minima were obtained. All calculated frequencies were scaled by a scaling factor 0.960 in order to correct the calculated value to experimentally observable.

### 3.4. Crystal Structure

The methodology for X-ray analysis [59,60,61] and additional information about the crystal are included in the Supplementary Materials. Crystal structure was deposited at the Cambridge Crystallographic Data Center, with deposit number CCDC 2,039,827, and is available free of charge via www.ccdc.cam.ac.uk/data_request/cif.

### 3.5. Biological Activity

#### 3.5.1. Cell Culture and Medium

The synthesized compounds and references were tested for cytotoxic activity in vitro against human cancer cell lines such as amelanotic melanoma (C-32, ATCC, Rockville, MD, USA), glioblastoma (SNB-19, DSMZ, Braunschweig, Germany), and breast cancer (T47D, and MDA-MB-231, ATCC, Rockville, MD, USA). Cell cultures were maintained using DMEM (Lonza, Basel, Switzerland) supplemented with 10% fetal bovine serum (FBS) (Biological Industries Cromwell, CT, USA) and penicillin (10,000 U/mL) –streptomycin (10 mg/mL) mix (Lonza, Basel, Switzerland).

#### 3.5.2. Cell Viability Studies

Cultures were performed in 96-well plates (Nunc Thermo Fisher Scientific, Waltham, MA, USA). Cells were seeded at 5 × 10^4^/well and incubated for 24 h (at 37 °C, 5% CO_2_, constant humidity). Then, the medium was replaced with a fresh one with the addition of test compounds in the concentration: 25; 12.5; 6.25; 3.12; 1.56; 0.78; 0.39; 0 µg/mL and incubated again for a further 72 h. After this time, the WST-1 test (Roche Molecular Biochemicals, Mannheim, Germany) was performed to assess the metabolic activity of the cells. The principle of the WST-1 colorimetric test is based on the ability of live cells to decompose bright red tetrazole salts of WST-1 to dark red formazan. The reaction is mediated by mitochondrial dehydrogenases and correlates with the number of cells and their metabolic activity. The absorbance was measured at λ = 450 nm with a UVM340 microplate reader (Biogenet, Józefów, Poland). Results were expressed as a mean value of at least three independent experiments performed in triplicate.

#### 3.5.3. Analysis of the Expression of H3, TP53, BCL-2, and BAX by qRT-PCR

The influence of tested compounds on the transcriptional activity of cells of the genes H3, TP53, BCL-2, and BAX was investigated. Assays were performed by real-time qRT-PCR on an Opticon TM DNA Engine (MJ Research, USA) using the QuantTect^®^ SYBR^®^ Green qRT-PCR Kit (Qiagen, Valencia, Spain). Cells were exposed to compound **7a** and **7b** for 24 h at a concentration equal to half of IC_50_ for appropriate cell line. For the reference compound, cisplatin, concentrations were 0.8 µg/mL (SNB-19 line) and 0.6 µg/mL (C-32 line). RNA extraction was performed on spin columns using the Quick RNATM MiniPrep kit (Zymo Research, Irvine, CA, USA). RNA extracts were assessed qualitatively and quantitatively. Total RNA integrity was checked electrophoretically on a 1.2% agarose gel with ethidium bromide. The amount and purity of total RNA in the extracts were determined spectrophotometrically with the HP8452A spectrophotometer (Hewlett Packard, Waldbronn, Germany).

#### 3.5.4. Statistical Analysis

All experiments were repeated in triplicate. Control treatment refers to untreated cells. As a positive control, used cells were treated with cisplatin. The data were expressed as mean ± standard deviation (SD). One-way ANOVA with post-hoc Tukey’s test was used to compare the data from different groups, *p* < 0.05 was considered statistically significant.

### 3.6. Physicochemical Descriptors (Lipophilicity, ADME Properties)

Chromatographic procedure by RP-TLC [62] is included in the Supplementary Materials. The ADME parameters for all derivatives were calculated using ACD/iLab software (Advanced Chemistry Development, Inc., Toronto, ON, Canada) [42].

### 3.7. Molecular Docking

The three-dimensional (3D) structures of studied compounds were generated in their low-energy conformation using Gaussian 16 (revision A.03) computer code [63] at the density functional theory (DFT, B3LYP) and 6–311 + G(d,p) basis sets. Target macromolecule for molecular docking studies was obtained from the Protein Data Bank (https://www.rcsb.org/). We used 3D crystal structures of EGFR (PDB ID: 1M17). Protein Preparation Wizard of UCSF Chimera (DockPrep) software [64] was used to process and prepare the protein. Missing chains were added, the implicit hydrogen atoms also were added to the atoms to satisfy their appropriate valences, and the ligand of no significance present in the protein structure was deleted. Then the structure was optimized by assigning the bond orders, bond angles, and topology. The formal atomic charges were fixed for the amino acid residues, and energy minimization was carried out.

Genetic Optimization for Ligand Docking (GOLD) 2020.1 [50] was used for the docking analysis. The region of interest used for GOLD docking was defined as the EGFR protein residues within the 10 Å of the reference erlotinib (X = 22.01, Y = 0.25, Z = 52.79 Å). For remaining water molecules, the “Toggle” option was on. Default values of all other parameters were used, and the complexes were submitted to 100 genetic algorithm runs using the ChemScore fitness function. After calculations, only the ten highest scored pose was returned as a docking result for ligand–cavity configuration. All obtained results were ranked according to their score value and presented in GOLD arbitrary units (a.u.). Calculation of protein–ligand binding free energy was performed using the K_DEEP_ predictor based on DCNNs (https://playmolecule.org/Kdeep) [52]. Molecular docking details were visualized using the BIOVIA Discovery Studio virtual environment [65] and Chimera [63]. Molecular dynamics simulation was performed with Nanoscale Molecular Dynamics software ver. 2.13 (NAMD, https://www.ks.uiuc.edu/Research/namd/) [57]. All input files were prepared using QwikMD [66] computer program based on GOLD output complexes. Protein–ligand systems have been solvated with 0.15 mol/L NaCl water box. Then system was minimized, annealed, and equilibrated. After that, 10 ns production simulation was performed. The MolAICal [56] is used to calculate the MM/GBSA between ligands and EGFR protein based on molecular dynamical (MD) simulated results by NAMD.

## 4. Conclusions

In the presented study, new 30-phosphate derivatives of betulin were synthesized, and evaluated as anticancer agents. Taking into account all the tested neoplastic cell lines, it can be seen that in the examined group of alkynyl derivatives, monoesters **7a**–**7e** (IC_50_ in range 0.76–15.40 µM) showed higher activity than diesters **6a**–**6e** (IC_50_ in range 5.65–31.46 µM). Compounds **7a** and **7b** were characterized by potent in vitro activity against the C-32 and SNB-16 cell lines. In addition, derivative **7a** characterized antitumor activity higher than that of the reference drug cisplatin against C-32, SND-19, and T47D cells. Comparing the activity of 28-propynoylbetulin with its 30-phosphate analogue **7a** in relation to appropriate tumor cell lines shows that the introduction of diethylphosphate substituent to the isopropenyl moiety resulted in an improvement in activity. With respect to T47D cells, alkynyl esters with 30-phosphate substituent (**7a**–**7c**) showed weaker activity than their phosphonate analogs (**11**–**13**). In relation to the SNB-16 and C-32 lines, no relationship between the activity and the type of the phosphorus substituent was observed.

The most active in vitro compounds, **7a** and **7b**, were tested on expression of H3, TP53, BAX, and BCL-2 genes. The results have shown that **7a** and **7b** can probably activate the apoptosis process in SNB-19 and C-32 cells. Further research is necessary to determine the exact path of cell death of the studied lines. The antitumor activity of triterpenes against the tested cell lines was more dependent on their chemical structure and less on lipophilicity. It was observed that the correlation between lipophilicity and antitumor activity was significant for the monoesters **7a**–**7e** containing a free hydroxyl group at the C3 position. The obtained results suggest that the relationship between ADME properties and antitumor activity is a complex issue.

Taking into account the potential mechanisms of antitumor activity, molecular docking of the most active in vitro derivatives, **7a** and **7b**, to the selected receptor, EGFR, was also carried out. In the library of designed phosphate derivatives of betulin, compounds **7a** and **7b** are reported to have good binding interactions as compared to standard erlotinib. Compound **7b** was found to have the highest binding affinity and form a perfect binding pose with EGFR, justified by KDEEP score. Furthermore, the molecular dynamic study of the most effective ligands **7a**,**b** has proved the stability of ligand–protein complexes with little deviation and fluctuation from 7 ns up to 10 ns. Compounds **7a** and **7b** demonstrated comparable degree of fit to the reference ligand erlotinib. Such a result may indicate a potential molecular target with which the antitumor activity of the test compounds is related.

## Figures and Tables

**Table 1:** Experimental band frequencies and assignments of Raman spectra for compounds **5**, **7a**, **7c**, **10**, **11**, and **13**.

Experimental Spectra (cm^−1^)						
5	7a	7c	10	11	13	
-	1719	1704	-	1715	1710	C=O stretching
1664	1652	1650	1625	1627	1629	C=C stretching in allyl/vinyl chains
1465–1450	1465–1450	1458	1465–1450	1465–1450	1458	CH_3_, CH_2_, CH bending in side chain and cycloalkane ring
-	1350	1367	1370	-	1360	P=O stretching, CH_3_, CH_2_, CH bending in side chain
1324	1325	1326	1325	1322	1334	
1284	1265	1275	-	-	-	P-O stretching in O=P-O-C moiety
-	-	-	1260	-	1261	P-C stretching in O=P-C=C moiety
1234	1235	1235	1237	1234	1234	P=O, CH_3_, CH_2_, CH bending in side chain and cycloalkane ring
-	1170	1187	1170	1173	1186 s	P-O stretching, CH_3_, CH_2,_ CH bending and C-C stretching in cycloalkane ring, C-O stretching
1143	1141	1145	-	-	1141	
1105	1097	1098	1100	1100	1101	
1038	1036	1044	1039	1039	1040	
921	921	922	-	-	-	P-O bending in O=P-O-C moiety
-	-	-	790	795	792	P-C bending in O=P-C=C moiety
-	755	766	-	752	752	O-C=O stretching, CH_3_, CH_2,_ CH bending in cycloalkane ring
736	745	732	735	735	735	CH_3_, CH_2,_ CH bending in cycloalkane ring
704	708	707	704	704	707	CH_3_, CH_2_ in side chain, P-O stretching
662	665	657	661	661	662	CH_3_, CH_2_, CH bending in cycloalkane ring, in side chains, stretching C-C in cycloalkane ring
614	618	614	619	618	620	
615	595	-	593	595	619	
560	558	-	561	560	560 w	
535	535	530	531	530	533 s	
460	459	470	560	460	462	stretching C-C in cycloalkane ring, C-H bending
414	410	402	414	410	413	
388	389	-	386	-	-	
232	234	240	233	-	236	OH bending

vw—very weak, w—weak, s—strong.

**Table 2:** Bond length and selected group Raman frequencies for the double bond C20–C29 experimental and calculated. Band shift (Δν_C=C_) was calculated as a difference between band frequencies for following sets of compounds: **5**–**10**, **7a**–**11**, and **7c**–**13**.

	Bond Length C20 = C29	Raman Spectra			
		Experimental Band Frequency (ν_C=C_)_exp_ [cm^−1^]	Shift_exp_ (Δν_C=C_)_exp_ [cm^−1^]	Calculated Band Frequency (ν_C=C_)_cal_ [cm^−1^]	Shift_cal_ (Δν_C=C_)_cal_ [cm^−1^]
**5**	1.326(5)	1664	39	1677	68
**10**	1.338(3)	1625		1609	
**7a**	-	1652	25	-	-
**11**	-	1627		-	-
**7c**	-	1650	21	-	-
**13**	-	1629		-	-

**Table 3:** Selected bond lengths (Å) for the crystal structure of 30-diethoxyphosphoryloxybetulin **5** and 28-cyclopropylpropynoyl-29-diethoxyphosphorylbetulin **13**.

Compound	Lengths of Selected Bonds [Å]		
	C20–C29	C20–C30	C20–C19
30-diethoxyphosphoryloxybetulin **5**	1.326(5)	1.490(5)	1.503(5)
28-cyclopropylpropynoyl- 29-diethoxyphosphorylbetulin **13** [7]	1.338(3)	1.515(3)	1.514(3)

**Table 4:** Effect of 30-phosphate derivatives of betulin and reference compounds (betulin and cisplatin) on the viability of cells from four cancer cell lines studied.

	Compound	Human Cell Line/IC_50_ ± SD [µM]			
		C-32	SNB-19	T47D	MDA-MB-231
**30-phosphate derivatives**	**4**	33.86 ± 1.59	5.01 ± 0.78	1.3 ± 0.13	14.48 ± 1.37
	**5**	43.46 ± 2.50	10.93 ± 1.33	1.04 ± 0.14	0.40 ± 0.02
	**6a**	---	---	9.50 ± 0.74	-
	**7a**	2.15 ± 0.22	0.91±0.09	2.61 ± 0.42	6.20 ± 0.22
	**6b**	NA	4.65 ± 0.51	NA	21.18 ± 1.24
	**7b**	0.80 ± 0.03	0.76 ± 0.14	15.40 ± 0.51	4.93 ± 0.44
	**6c**	NA	NA	NA	NA
	**7c**	11.90 ± 0.52	6.29 ± 0.12	NA	9.11 ± 0.39
	**6d**	31.46 ± 1.56	11.22 ± 0.28	NA	NA
	**7d**	6.90 ± 0.40	4.38 ± 0.04	9.47 ± 0.03	NA
	**6e**	11.43 ± 0.76	9.23 ± 0.57	NA	---
	**7e**	3.97 ± 0.86	1.59 ± 0.09	NA	---
**29-phosphonate derivatives**	10 *	8.80 ± 0.17	10.20 ± 0.69	10.71 ± 0.86	---
	11 *	0.60 ± 0.08	0.43 ± 0.14	0.70 ± 0.13	---
	12 *	7.70 ± 0.11	11.67 ± 0.44	14.94 ± 0.90	---
	13 *	6.99±1.07	---	12.2±0.84	---
	14 *	9.00 ± 0.53	---	NA	---
	**Betulin**	15.61 ± 1.11	9.93 ± 0.25	NA	7.76 ± 1.87
	**Cisplatin**	4.29 ± 0.65	5.29 ± 0.12	6.26 ± 1.24	5.47 ± 0.05

NA means IC_50_ > 50 µM. * Phosphonate derivatives of betulin; synthesis and the results of the anticancer activity test were described previously [7].

**Table 5:**
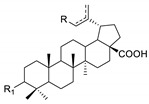
Effect of 30-phosphate derivatives of betulonic acid **8**, betulinic acid **9**, and reference compounds on the viability of cells from two cancer cell lines studied (C-32 and SNB-19).

R		30-OP(O)(OC_2_H_5_)_2_		29-P(O)(OC_2_H_5_)_2_ *		30-P(O)(OC_2_H_5_)_2_ *		H
**R_1_**		O= Comp. **8**	OH Comp. **9**	O=	OH	CH_3_C(O)O	OH	OH
IC_50_[µM]	C-32	45.74 ± 6.85	NA	45.52 ± 3.72	47.46 ± 2.96	30.74 ± 2.17	NA	8.63 ± 0.90
	SNB-19	45.75 ± 5.27	5.98 ± 0.43	1.64 ± 0.18	7.67 ± 1.57	1.29 ± 0.15	NA	20.09 ± 1.26

*—Phosphonate derivatives of betulonic and betulinic acids; synthesis and the results of the anti-HIV-1 activity test were described previously [9].NA Means IC_50_ > 50 µM.

**Table 6:** The experimental values of lipophilicity for tested compounds **3**–**5**, **6a**–**6e**, and **7a**–**7e**.

Compound	R_M0_	-b	*r*	logP_TLC_
**3**	5.655	0.064	0.997	7.362
**4**	5.740	0.064	0.998	7.470
**5**	3.719	0.046	0.984	4.914
**6a**	6.893	0.080	0.977	8.929
**7a**	5.318	0.062	0.988	6.937
**6b**	6.884	0.078	0.994	8.917
**7b**	5.431	0.063	0.994	7.079
**6c**	6.419	0.071	0.998	8.330
**7c**	5.587	0.064	0.985	7.277
**6d**	7.877	0.086	0.998	10.174
**7d**	6.145	0.070	0.992	7.982
**6e**	6.490	0.076	0.994	8.419
**7e**	5.302	0.063	0.993	6.916

**Table 7:** The correlation between the cytotoxicity (IC_50_) and the log P values for monoesters **7a**–**7e**.

Compounds	Cell Line	Equation	*r*
**7a–7e**	C-32	IC_50_ = −21.887LogP^2^ + 331.21LogP—1242.1 (A)	0.720
**7a–7e**	SNB-19	IC_50_ = −12.262LogP^2^ + 186.35LogP—701.71 (B)	0.826

**Table 8:** Prediction of ADME properties of compounds **3**–**5**, **6a**–**6e**, and **7a**–**7e**.

Compound	M	logBB	nHBD	nHBA	nROTB	tPSA
**3**	542.80	0.07	1	5	7	72.83
**4**	678.89	−0.55	0	8	13	107.17
**5**	594.81	−0.32	2	6	9	95.03
**6a**	698.88	−1.70	0	8	15	107.17
**7a**	646.85	−0.85	1	7	12	101.10
**6b**	726.93	−1.40	0	8	15	107.17
**7b**	660.87	−0.79	1	7	12	101.10
**6c**	779.01	−1.70	0	7	17	90.10
**7c**	686.91	−0.85	1	7	13	101.10
**6d**	851.07	−2.00	0	7	17	90.10
**7d**	722.94	−1.40	1	7	13	101.10
**6e**	758.93	−1.70	0	10	19	125.63
**7e**	676.87	−0.85	1	8	14	110.33

**Table 9:** The top-ranked ChemScore fitness score function obtained with GOLD and binding energy obtained with K_DEEP_ for betulin derivatives **7a** and **7b**.

Compound	GOLD [a.u.]	K_DEEP_ ΔG [kcal/mol]
**7a**	30.87	−10.29
**7b**	31.33	−11.48
erlotinib	31.06	−9.63 (−8.93 *)

* Calculating for crystal structure of 1M17.

**Table 10:** Calculated binding free energies of tested compounds.

Compound	ΔE_(internal)_	ΔE_(electrostatic)_ + ΔG_(sol)_	ΔE_(VDW)_ + ΔG_(sol_)	ΔG_(bind)_
**7a**	−4.96	20.25	−61.82	−46.53
**7b**	−5.91	24.88	−62.48	−43.51
erlotinib	−7.40	11.80	−35.18	−23.38

## Data Availability

Not applicable.

## Supplementary Materials

Synthesis—Materials and methods, Figure S1a. ^1^H NMR, 3,28-diacetoxy-30-diethoxyphosphoryloxybetulin **4**, Figure S1b. ^13^C NMR, 3,28-diacetoxy-30-diethoxyphosphoryloxybetulin **4**, Figure S1c. ^31^P NMR, 3,28-diacetoxy-30-diethoxyphosphoryloxybetulin **4**, Figure S1d. HR-MS, 3,28-diacetoxy-30-diethoxyphosphoryloxybetulin **4**, Figure S2a. ^1^H NMR, 30-diethoxyphosphoryloxybetulin **5**, Figure S2b. ^13^C NMR, 30-diethoxyphosphoryloxybetulin **5**, Figure S2c. ^31^P NMR, 30-diethoxyphosphoryloxybetulin **5**, Figure S2d. HR-MS, 30-diethoxyphosphoryloxybetulin **5**, Figure S3a. ^1^H NMR, 30-diethoxyphosphoryloxy-28-propynoylbetulin **7a**, Figure S3b. ^13^C NMR, 30-diethoxyphosphoryloxy-28-propynoylbetulin **7a**, Figure S3c. ^31^P NMR, 30-diethoxyphosphoryloxy-28-propynoylbetulin **7a**, Figure S3d. HR-MS, 30-diethoxyphosphoryloxy-28-propynoylbetulin **7a**, Figure S4a. ^1^H NMR, 28-(2-butynoyl)-30-diethoxyphosphoryloxybetulin **7b**, Figure S4b. ^13^C NMR, 28-(2-butynoyl)-30-diethoxyphosphoryloxybetulin **7b**, Figure S4c. ^31^P NMR, 28-(2-butynoyl)-30-diethoxyphosphoryloxybetulin **7b**, Figure S4d. HR-MS, 28-(2-butynoyl)-30-diethoxyphosphoryloxybetulin **7b**, Figure S5a. ^1^H NMR, 28-(2-cyclopropylpropynoyl)-30-diethoxyphosphoryloxybetulin **7c**, Figure S5b. ^13^C NMR, 28-(2-cyclopropylpropynoyl)-30-diethoxyphosphoryloxybetulin **7c**, Figure S5c. ^31^P NMR, 28-(2-cyclopropylpropynoyl)-30-diethoxyphosphoryloxybetulin **7c**, Figure S5d. HR-MS, 28-(2-cyclopropylpropynoyl)-30-diethoxyphosphoryloxybetulin **7c**, Table S1. Calculated Raman band frequencies and assignments for compounds **5** and **10**, X-ray diffraction experiment, Table S2 Crystal parameters, data collection and refinement details for compound **5**, Table S3 Monocrystal characteristic, Table S4 Selected hydrogen bonds in the crystal structure of compound **5**, Chromatographic procedure by RP-TLC, Table S5. The theoretical values of lipophilicity for tested compounds **3**–**5**, **6a**–**6e** and **7a**–**7e**, Table S6. The correlation matrix for theoretically obtained lipophilicity parameters of compounds **3**–**5**, **6a**–**6e** and **7a**–**7e**.
