# Supplementary material for: Molecular Structure, In Vitro Anticancer Study and Molecular Docking of New Phosphate Derivatives of Betulin
Source: Molecules. 2021 Jan 31;26(3):737. doi: 10.3390/molecules26030737 (PMC7866984; doi:10.3390/molecules26030737)
Supplement: Supplementary file 1 [file molecules-26-00737-s001.pdf]

molecules

Supplementary materials

Molecular structure, *in vitro* anticancer study and molecular docking of new phosphate derivatives of betulin

Elwira Chrobak[1], Maria Jastrzębska[2], Ewa Bębenek[1], Monika Kadela-Tomanek[1], Krzysztof Marciniec[1], Małgorzata Latocha[3], Roman Wrzalik[2], Joachim Kusz[4], Stanisław Boryczka[1]*

Department of Organic Chemistry, Faculty of Pharmaceutical Sciences in Sosnowiec, Medical University of Silesia, Katowice, 4 Jagiellońska Str., 41-200 Sosnowiec, Poland

[2] Silesian Center for Education and Interdisciplinary Research, University of Silesia, Institute of Physics, 75 Pułku Piechoty 1a, 41-500 Chorzów, Poland

[3] Department of Cell Biology, Faculty of Pharmaceutical Sciences in Sosnowiec, Medical University of Silesia, Katowice, 8 Jedności Str., 41-200 Sosnowiec, Poland

[4] University of Silesia, Institute of Physics, 75 Pułku Piechoty Str., 41 500 Chorzów, Poland

*Corresponding author.

E-mail address: echrobak@sum.edu.pl

LIST OF CONTENTS

Synthesis - ***Materials and methods***………………………………………………………. 3

Nuclear Magnetic Resonance spectra for selected compounds

Fig. S1a. ^{1}H NMR, 3,28-diacetoxy-30-diethoxyphosphoryloxybetulin **4**………………... 4

Fig. S1b. ^{13}C NMR, 3,28-diacetoxy-30-diethoxyphosphoryloxybetulin **4** ……………… 4

Fig. S1c. ^{31}P NMR, 3,28-diacetoxy-30-diethoxyphosphoryloxybetulin **4**………………... 5

Fig. S1d. HR-MS, 3,28-diacetoxy-30-diethoxyphosphoryloxybetulin **4**………………… 5

Fig. S2a. ^{1}H NMR, 30-diethoxyphosphoryloxybetulin **5**……………………………... 6

Fig. S2b. ^{13}C NMR, 30-diethoxyphosphoryloxybetulin **5**……………………………... 6

Fig. S2c. ^{31}P NMR, 30-diethoxyphosphoryloxybetulin **5**…………………………..….. 7

Fig. S2d. HR-MS, 30-diethoxyphosphoryloxybetulin **5**………………………………….. 7

Fig. S3a. ^{1}H NMR, 30-diethoxyphosphoryloxy-28-propynoylbetulin **7a**………………... 8

Fig. S3b. ^{13}C NMR, 30-diethoxyphosphoryloxy-28-propynoylbetulin **7a**……………..… 8

Fig. S3c. ^{31}P NMR, 30-diethoxyphosphoryloxy-28-propynoylbetulin **7a**……………….. 9

Fig. S3d. HR-MS, 30-diethoxyphosphoryloxy-28-propynoylbetulin **7a**………………… 9

Fig. S4a. ^{1}H NMR, 28-(2-butynoyl)-30-diethoxyphosphoryloxybetulin **7b**…………….. 10

Fig. S4b. ^{13}C NMR, 28-(2-butynoyl)-30-diethoxyphosphoryloxybetulin **7b**…………… 10

Fig. S4c. ^{31}P NMR, 28-(2-butynoyl)-30-diethoxyphosphoryloxybetulin **7b**…………….. 11

Fig. S4d. HR-MS, 28-(2-butynoyl)-30-diethoxyphosphoryloxybetulin **7b**……………… 11

Fig. S5a. ^{1}H NMR, 28-(2-cyclopropylpropynoyl)-30-diethoxyphosphoryloxybetulin **7c**.. 12

Fig. S5b. ^{13}C NMR, 28-(2-cyclopropylpropynoyl)-30-diethoxyphosphoryloxybetulin **7c**. 12

Fig. S5c. ^{31}P NMR, 28-(2-cyclopropylpropynoyl)-30-diethoxyphosphoryloxybetulin **7c**.. 13

Fig. S5d. HR-MS, 28-(2-cyclopropylpropynoyl)-30-diethoxyphosphoryloxybetulin **7c**… 13

Table S1. Calculated Raman band frequencies and assignments for compounds **10** and **5**. 14

X-ray diffraction experiment……………………………………………………… 15

Table S2 Crystal parameters, data collection and refinement details for compound **5**….. 16

Table S3 Monocrystal characteristic………………………………………………….. 16

Table S4 Selected hydrogen bonds in the crystal structure of compound **5**……………… 17

Chromatographic procedure by RP-TLC…………………………………………… 17

Table S5. The theoretical values of lipophilicity for tested compounds **3-5**, **6a-6e** and **7a-7e**……………………………………………………………………….… 18

Table S6. The correlation matrix for theoretically obtained lipophilicity parameters of compounds **3-5, 6a-6e** and **7a-7e**……………………………………………………. 18

Synthesis - *Materials and methods*

In the synthesis, commercially available reagents were used (Sigma-Aldrich, Saint Louis, MO, USA). The solvents were purified according to the standard methods. The NMR spectra (^{1}H, 600 MHz; ^{13}C, 150 MHz; ^{31}P, 243 MHz) were recorded on a Bruker AVANCE III HD 600 spectrometer in deuterated chloroform ($CDCl_3$). Chemical shifts (δ) values were reported in parts per million (ppm) relative to the solvent signal as internal standard. The coupling constants (*J*) are expressed in Hertz (Hz). Calculation of the exact molecular mass for new compounds was performed using "The Exact Mass Calculator, Single Isotope Version" (http://www.sisweb.com/referenc/tools/exactmass.htm). High-resolution mass (HR-MS) spectra were recorded with Bruker Impact II. Melting points were uncorrected and measured by Electrothermal IA 9300 melting point apparatus. The progress of reaction was monitored by TLC (silica gel 60 254F plates, Merck). A mixture of dichloromethane/ethanol (40:1 or 15:1, v/v) or hexane/ethyl acetate (3:2, v/v) was used as a developing system. The spots were visualized by spraying with a solution of 5% sulfuric acid and heating to 100˚C. The compounds were purified by column chromatography (silica gel 60, <63 µm, Merck) in the appropriate solvent system.

Fig. S1a. ^{1}H NMR, 3,28-diacetoxy-30-diethoxyphosphoryloxybetulin **4**

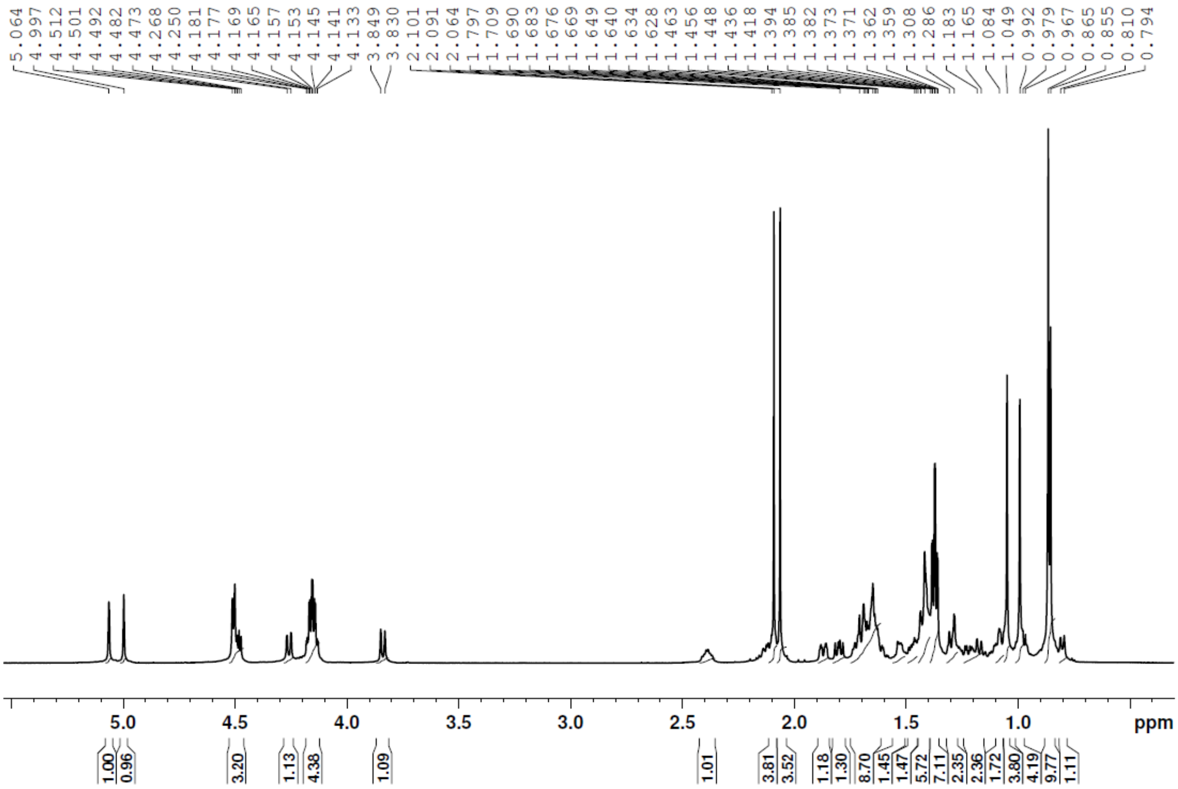

Fig. S1b. ^{13}C NMR, 3,28-diacetoxy-30-diethoxyphosphoryloxybetulin **4**

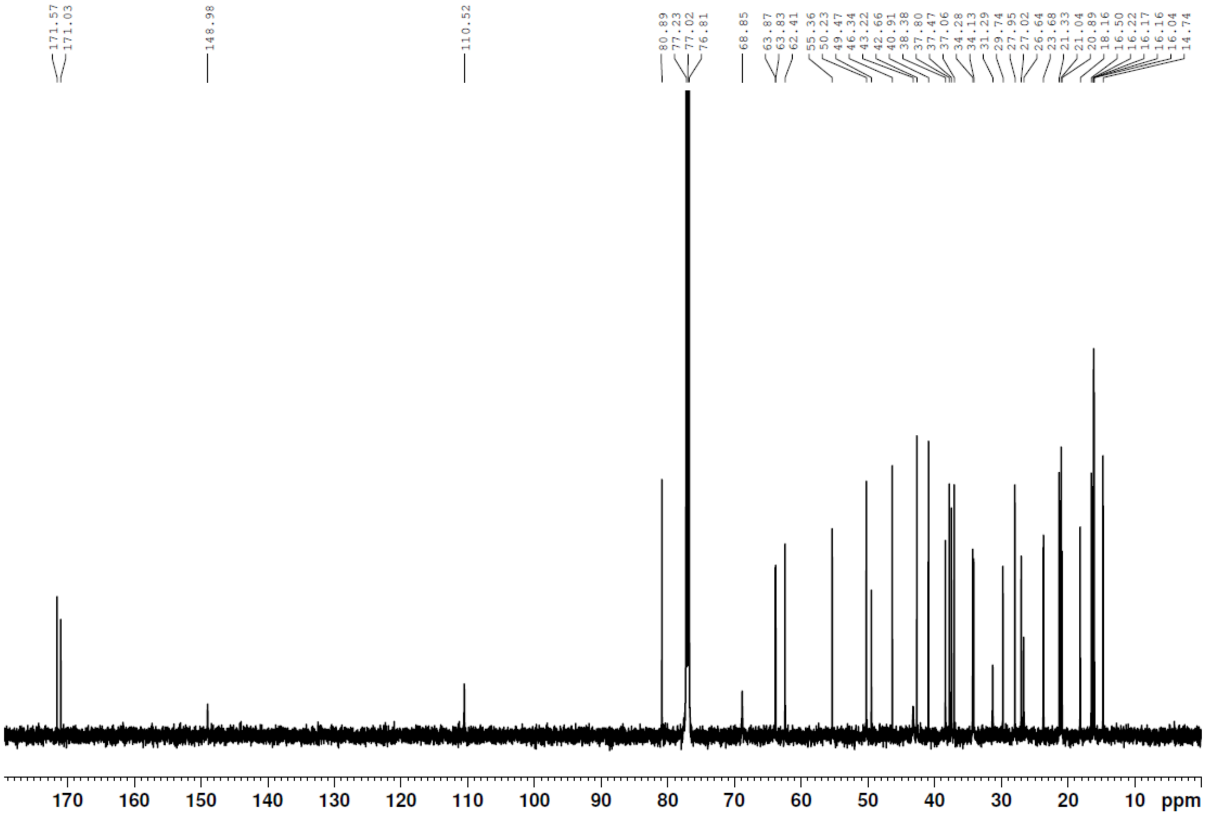

Fig. S1c. ^{31}P NMR, 3,28-diacetoxy-30-diethoxyphosphoryloxybetulin **4**

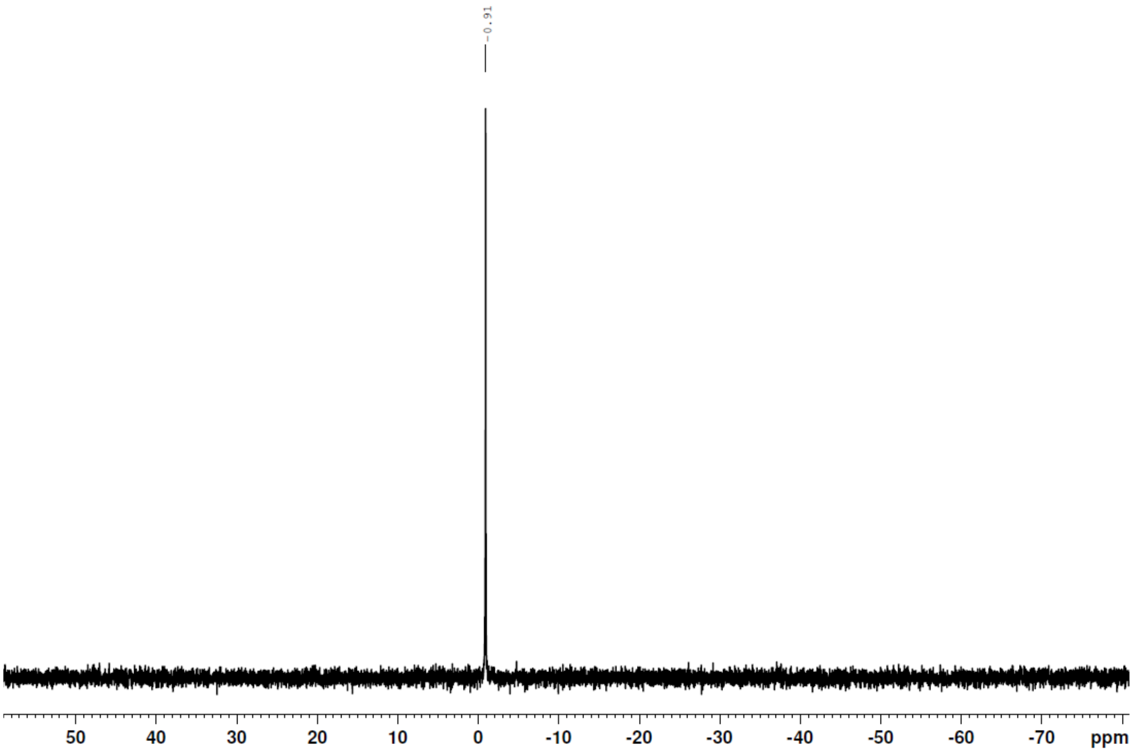

Fig. S1d. HR-MS, 3,28-diacetoxy-30-diethoxyphosphoryloxybetulin **4**

Mass Spectrum List Report

Analysis Info

Analysis Name	D:\Data\PM_20171204\ECh 91.d	Acquisition Date	5/29/2018 11:56:50 AM
Method	low_mass.m	Operator	KM
Sample Name	AN 40_18	Instrument	impact II 1825265.10082
Comment			

Acquisition Parameter

Source Type	APCI	Ion Polarity	Negative	Set Nebulizer	2.0 Bar
Focus	Active	Set Capillary	4000 V	Set Dry Heater	200 °C
Scan Begin	100 m/z	Set End Plate Offset	-500 V	Set Dry Gas	5.0 l/min
Scan End	2000 m/z	Set Charging Voltage	2000 V	Set Divert Valve	Source
		Set Corona	2000 nA	Set APCI Heater	450 °C

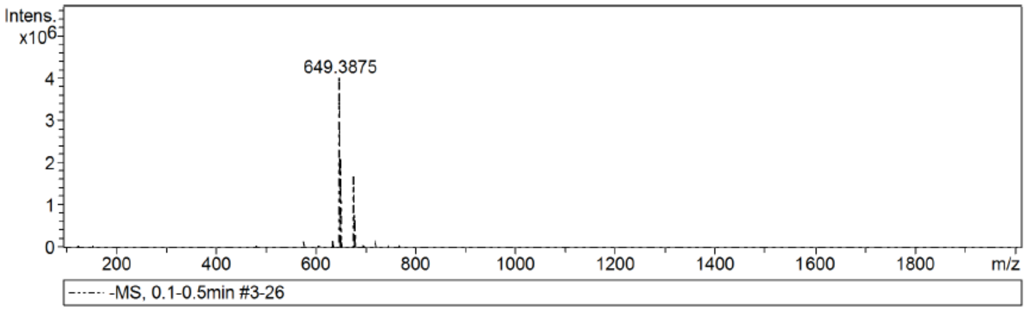

#	m/z	Res.	S/N	I	I %	FWHM
1	649.3875	34211	93549.7	4005252	100.0	0.0190
2	677.4180	47699	39267.9	1700025	42.4	0.0142

Fig. S2a. ^{1}H NMR, 30-diethoxyphosphoryloxybetulin **5**

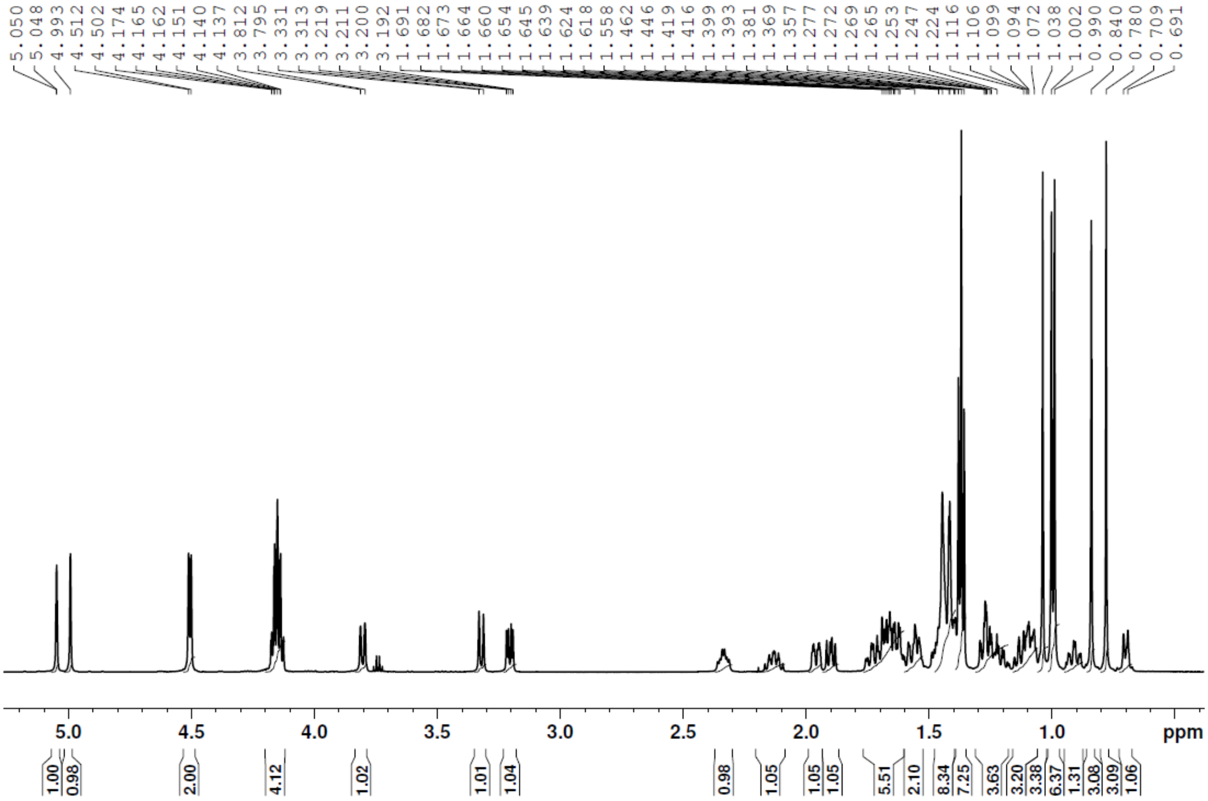

Fig. S2b. ^{13}C NMR, 30-diethoxyphosphoryloxybetulin **5**

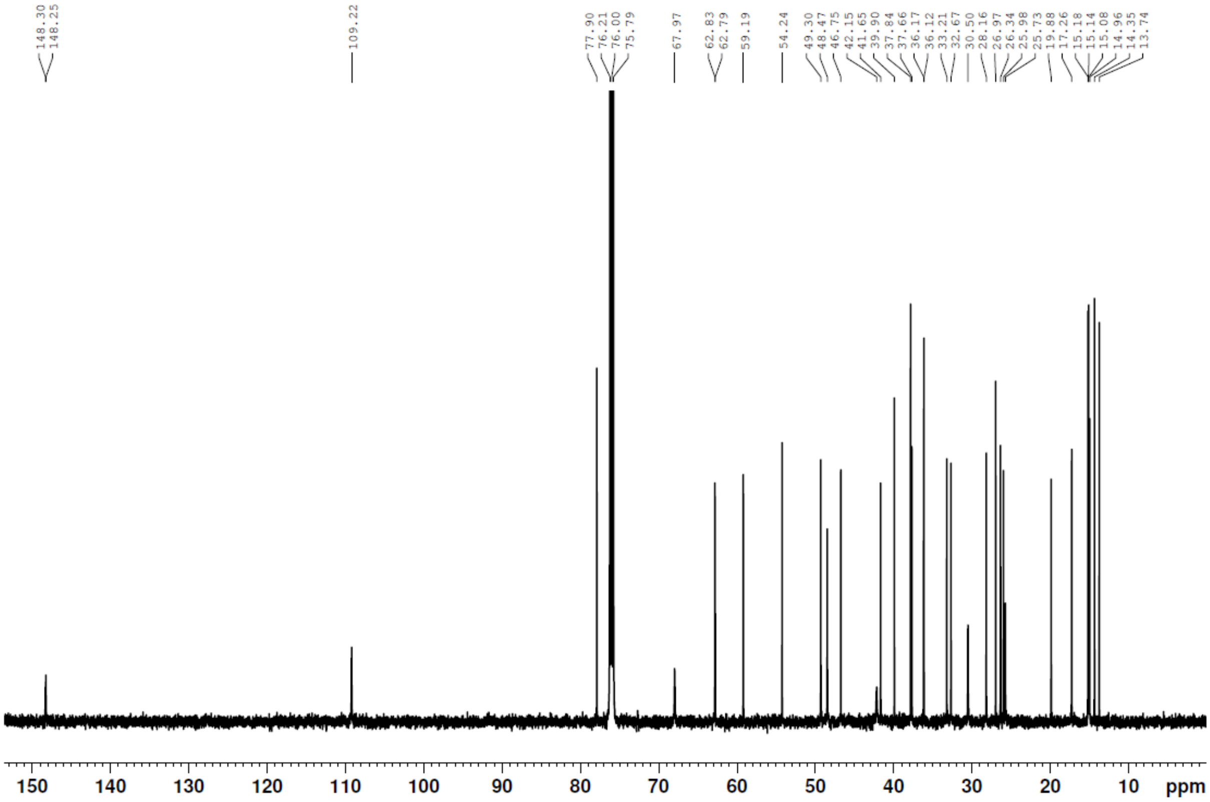

Fig. S2c. ^{31}P NMR, 30-diethoxyphosphoryloxybetulin **5**

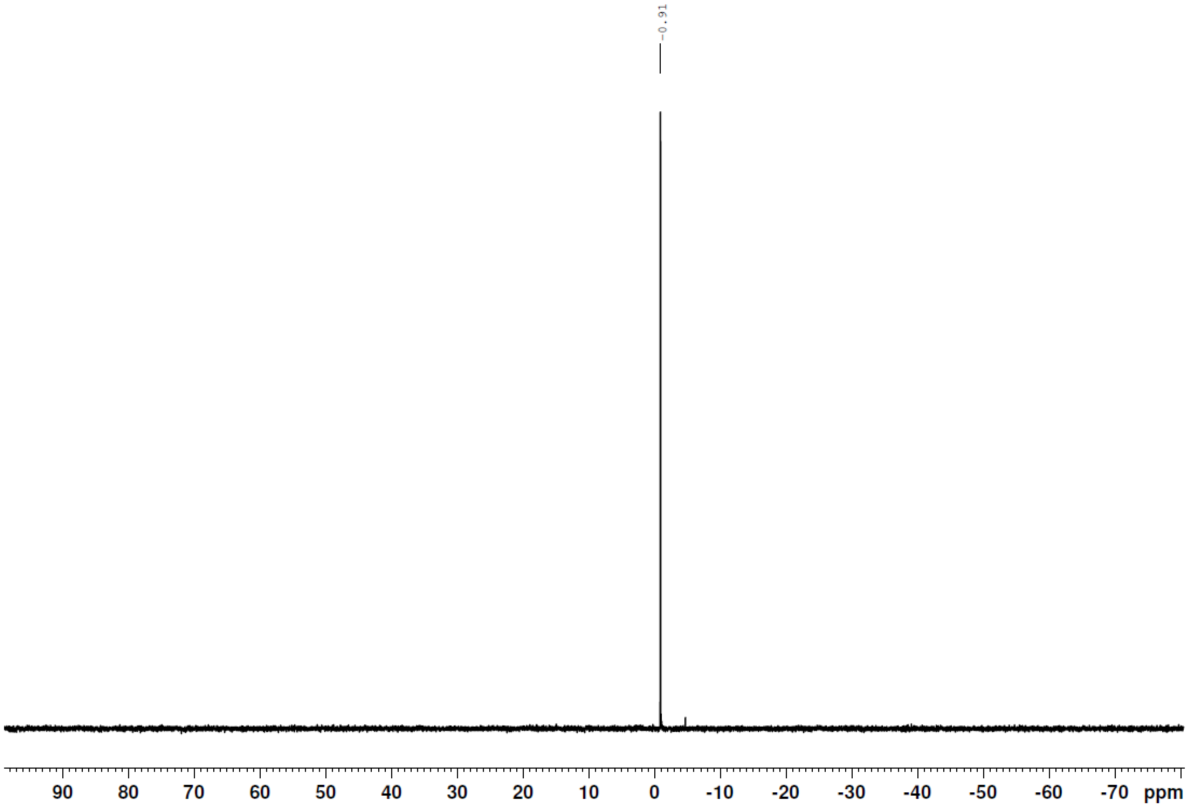

Fig. S2d. HR-MS, 30-diethoxyphosphoryloxybetulin **5**

Mass Spectrum List Report

Analysis Info

Analysis Name	D:\Data\PM_20171204\ECh 156.d	Acquisition Date	5/29/2018 12:00:10 PM
Method	low_mass.m	Operator	KM
Sample Name	AN 40_18	Instrument	impact II 1825265.10082
Comment			

Acquisition Parameter

Source Type	APCI	Ion Polarity	Negative	Set Nebulizer	2.0 Bar
Focus	Active	Set Capillary	4000 V	Set Dry Heater	200 °C
Scan Begin	100 m/z	Set End Plate Offset	-500 V	Set Dry Gas	5.0 l/min
Scan End	2000 m/z	Set Charging Voltage	2000 V	Set Divert Valve	Source
		Set Corona	2000 nA	Set APCI Heater	450 °C

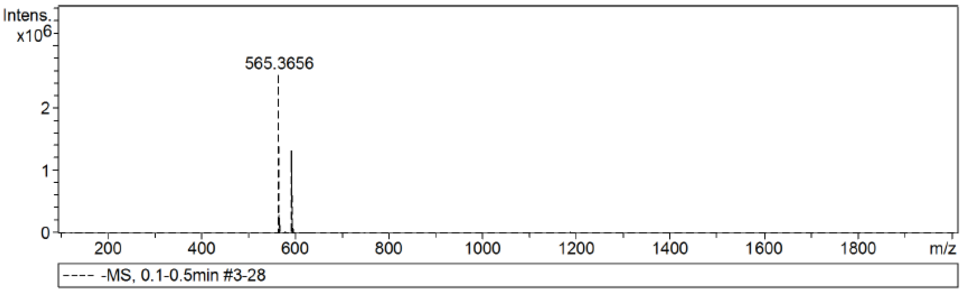

#	m/z	Res.	S/N	I	I %	FWHM
1	565.3656	32462	104071.4	2548734	100.0	0.0174
2	593.3959	43911	48052.0	1265967	49.7	0.0135

Fig. S3a. ^{1}H NMR, 30-diethoxyphosphoryloxy-28-propynoylbetulin **7a**

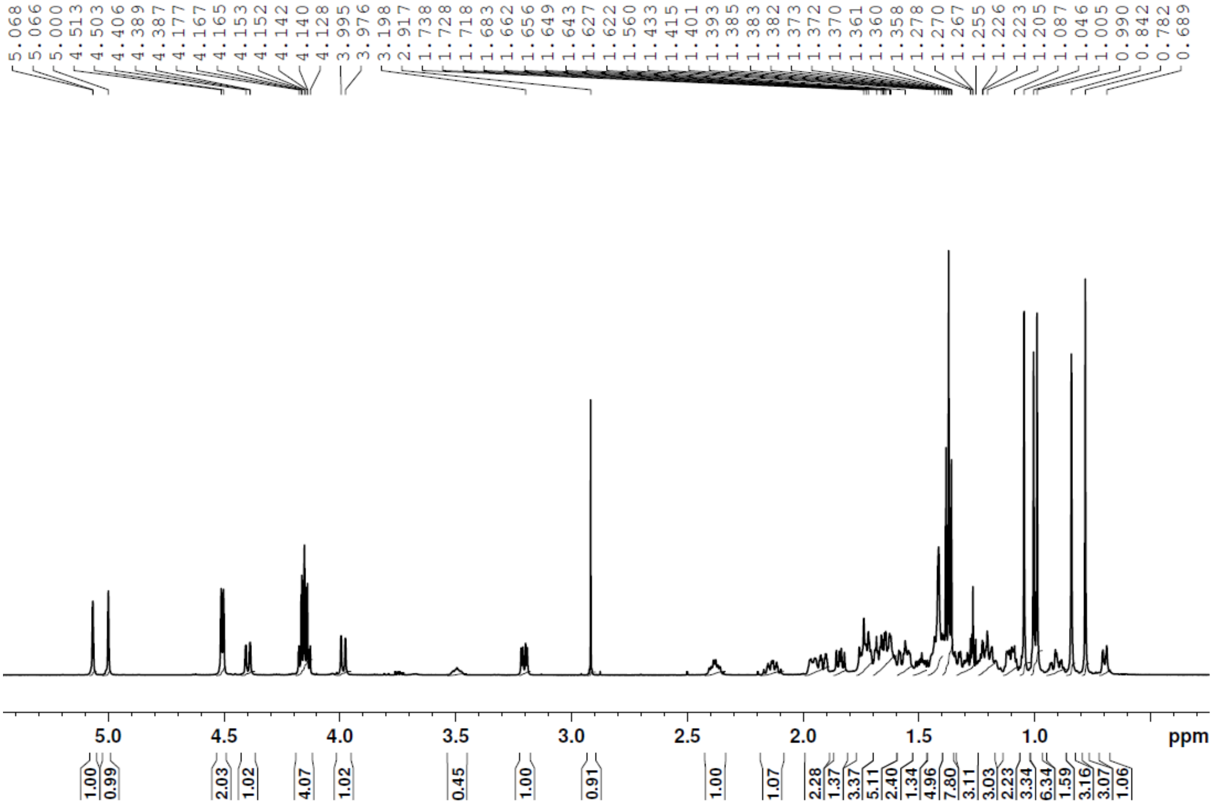

Fig. S3b. ^{13}C NMR, 30-diethoxyphosphoryloxy-28-propynoylbetulin **7a**

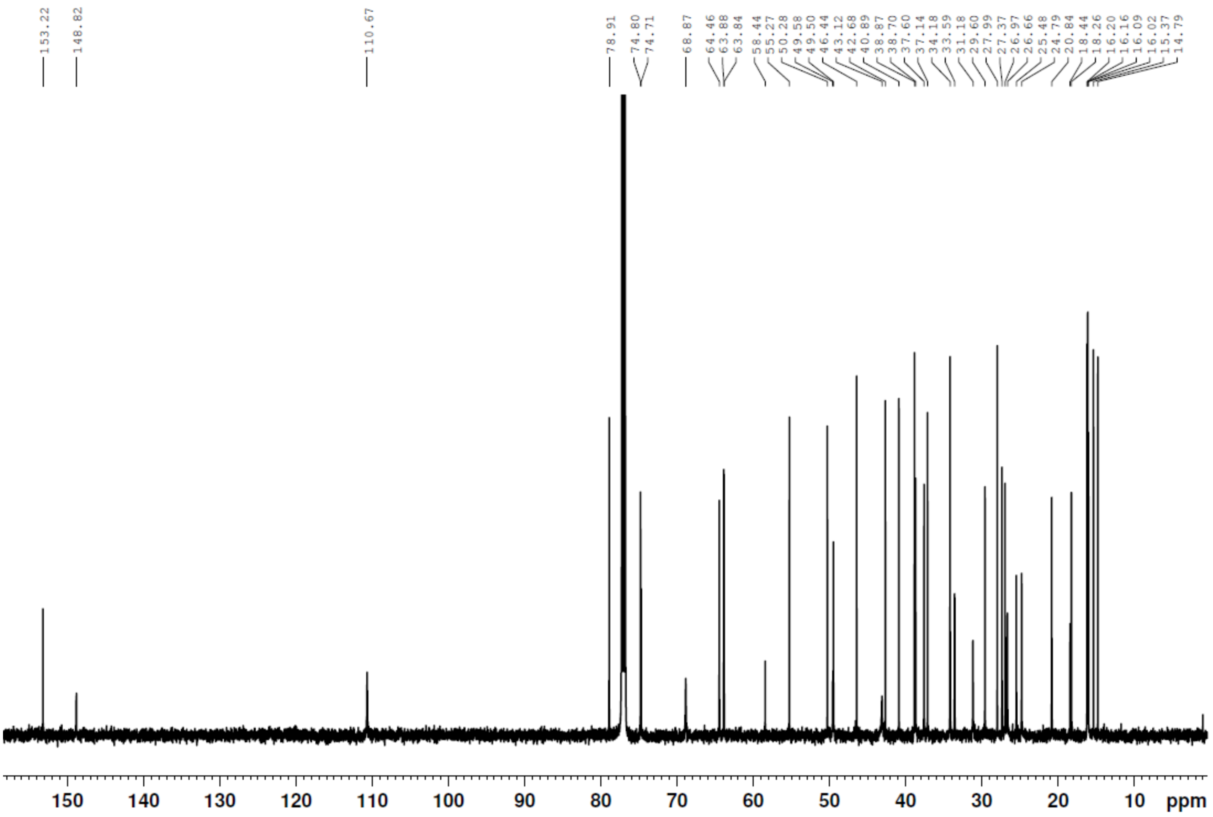

Fig. S3c. ^{31}P NMR, 30-diethoxyphosphoryloxy-28-propynoylbetulin **7a**

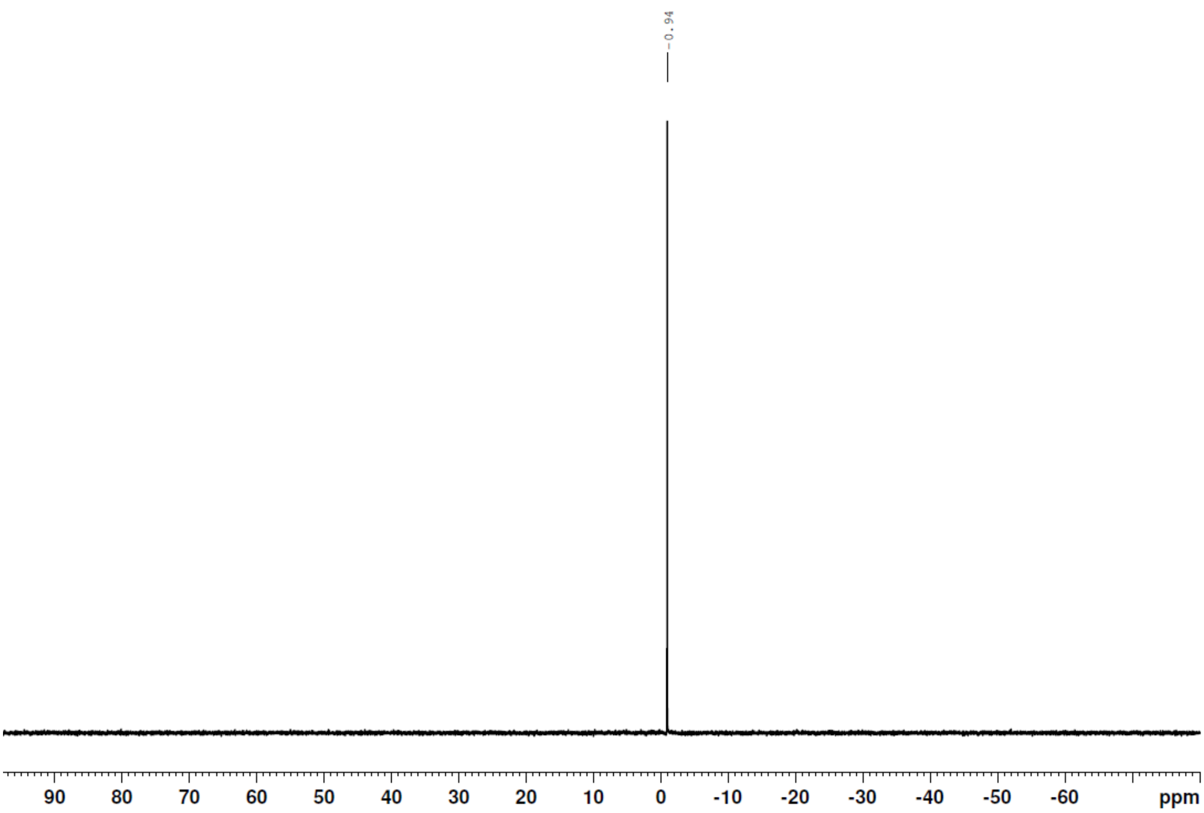

Fig. S3d. HR-MS, 30-diethoxyphosphoryloxy-28-propynoylbetulin **7a**

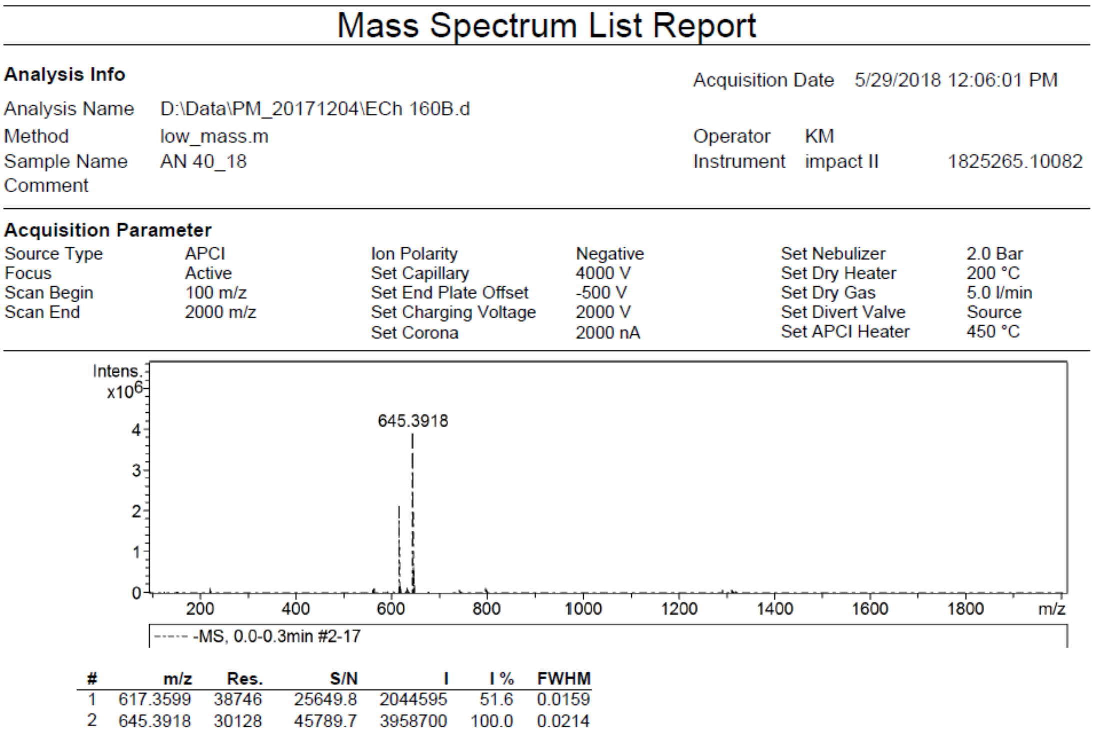
Mass Spectrum List Report

Analysis Info

Analysis Name	D:\Data\PM_20171204\ECh 160B.d	Acquisition Date	5/29/2018 12:06:01 PM
Method	low_mass.m	Operator	KM
Sample Name	AN 40_18	Instrument	impact II 1825265.10082
Comment			

Acquisition Parameter

Source Type	APCI	Ion Polarity	Negative	Set Nebulizer	2.0 Bar
Focus	Active	Set Capillary	4000 V	Set Dry Heater	200 °C
Scan Begin	100 m/z	Set End Plate Offset	-500 V	Set Dry Gas	5.0 l/min
Scan End	2000 m/z	Set Charging Voltage	2000 V	Set Divert Valve	Source
		Set Corona	2000 nA	Set APCI Heater	450 °C

#	m/z	Res.	S/N	I	I %	FWHM
1	617.3599	38746	25649.8	2044595	51.6	0.0159
2	645.3918	30128	45789.7	3958700	100.0	0.0214

Fig. S4a. ^{1}H NMR, 28-(2-butynoyl)-30-diethoxyphosphoryloxybetulin **7b**

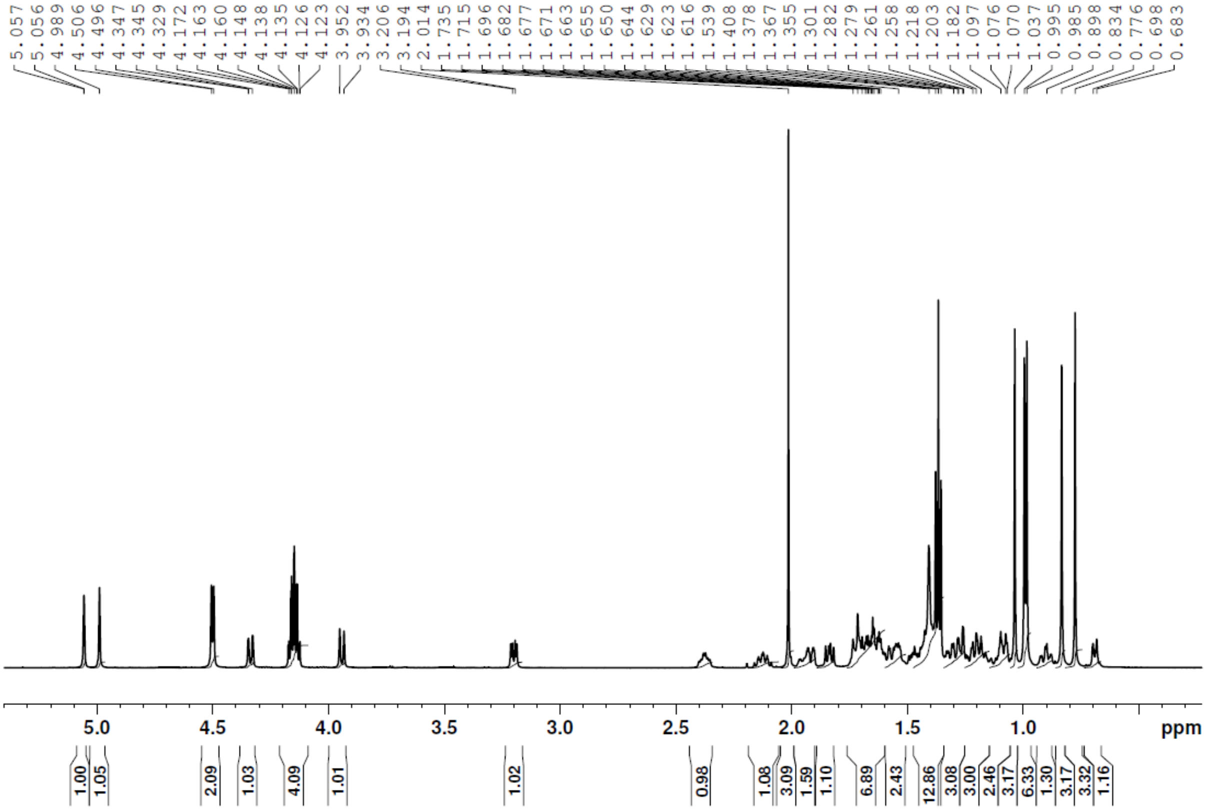

Fig. S4b. ^{13}C NMR, 28-(2-butynoyl)-30-diethoxyphosphoryloxybetulin **7b**

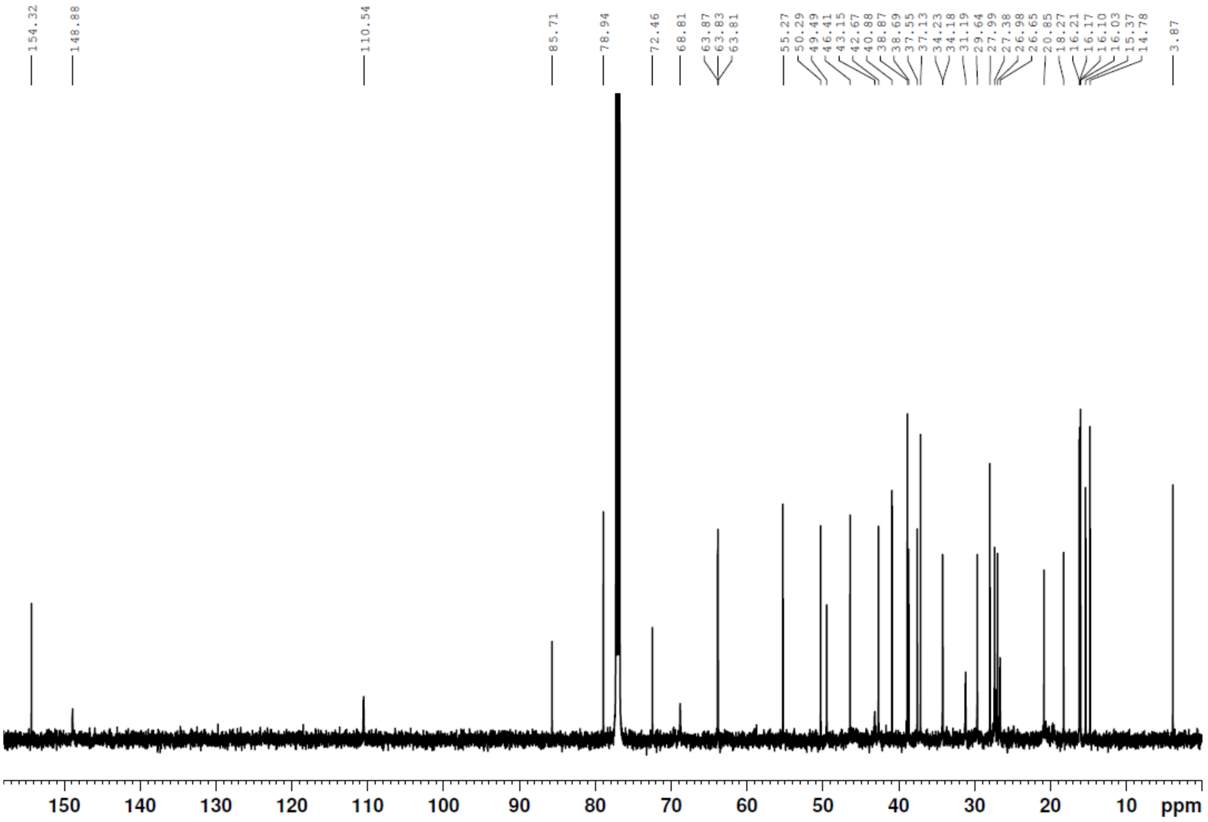

Fig. S4c. ^{31}P NMR, 28-(2-butynoyl)-30-diethoxyphosphoryloxybetulin **7b**

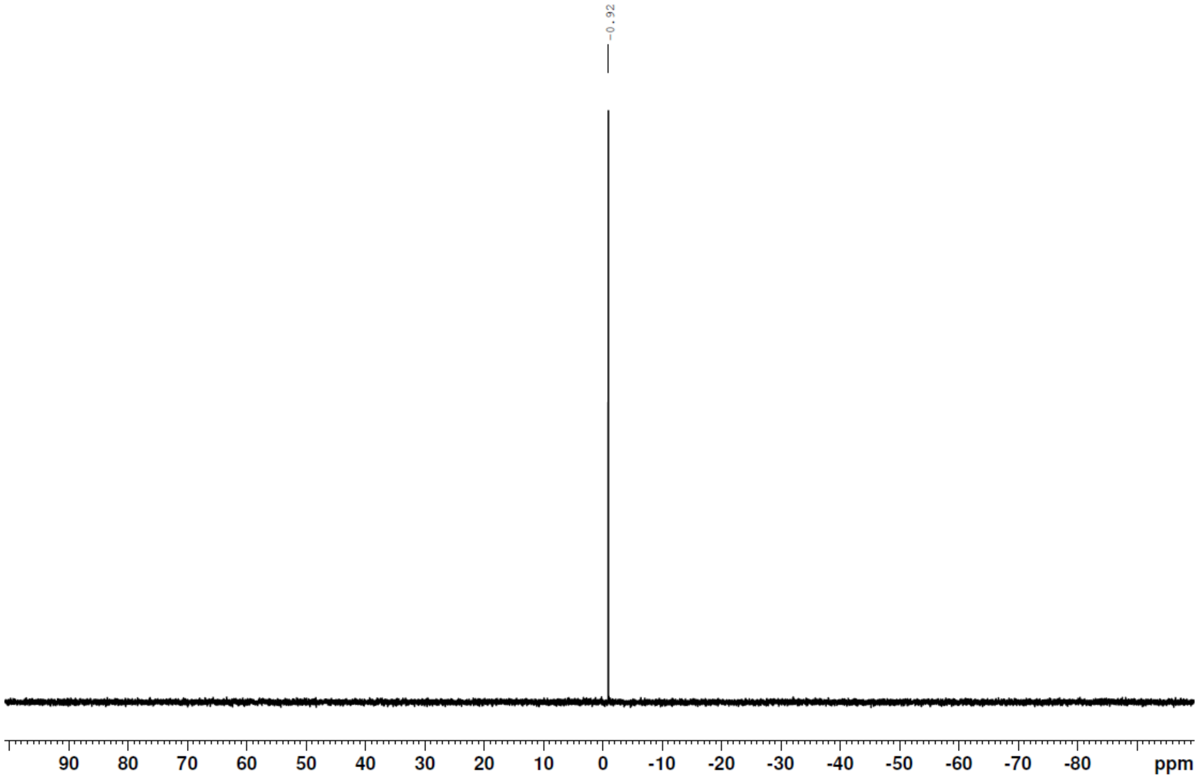

Fig. S4d. HR-MS, 28-(2-butynoyl)-30-diethoxyphosphoryloxybetulin **7b**

Mass Spectrum List Report

Analysis Info

Analysis Name	D:\Data\PM_20171204\ECh 197B.d	Acquisition Date	5/29/2018 12:25:52 PM
Method	low_mass.m	Operator	KM
Sample Name	AN 40_18	Instrument	impact II 1825265.10082
Comment			

Acquisition Parameter

Source Type	APCI	Ion Polarity	Negative	Set Nebulizer	2.0 Bar
Focus	Active	Set Capillary	4000 V	Set Dry Heater	200 °C
Scan Begin	100 m/z	Set End Plate Offset	-500 V	Set Dry Gas	5.0 l/min
Scan End	2000 m/z	Set Charging Voltage	2000 V	Set Divert Valve	Source
		Set Corona	2000 nA	Set APCI Heater	450 °C

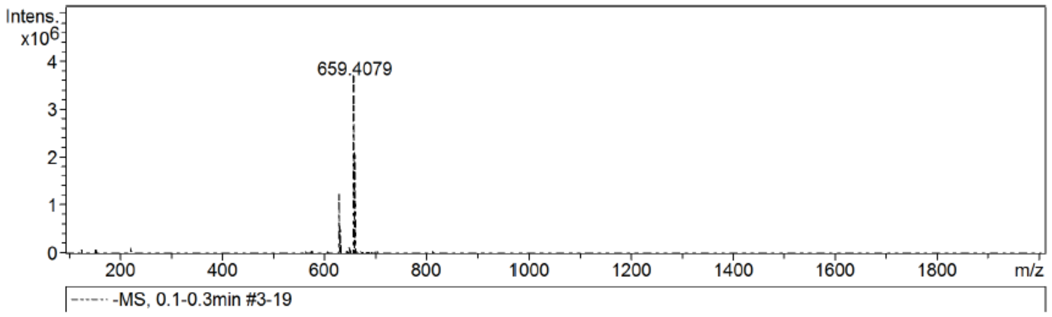

#	m/z	Res.	S/N	I	I %	FWHM
1	631.3761	42825	23532.0	1261227	35.0	0.0147
2	659.4079	32459	63725.6	3608503	100.0	0.0203

Fig. S5a. ^{1}H NMR, 28-(2-cyclopropylpropynoyl)-30-diethoxyphosphoryloxybetulin **7c**

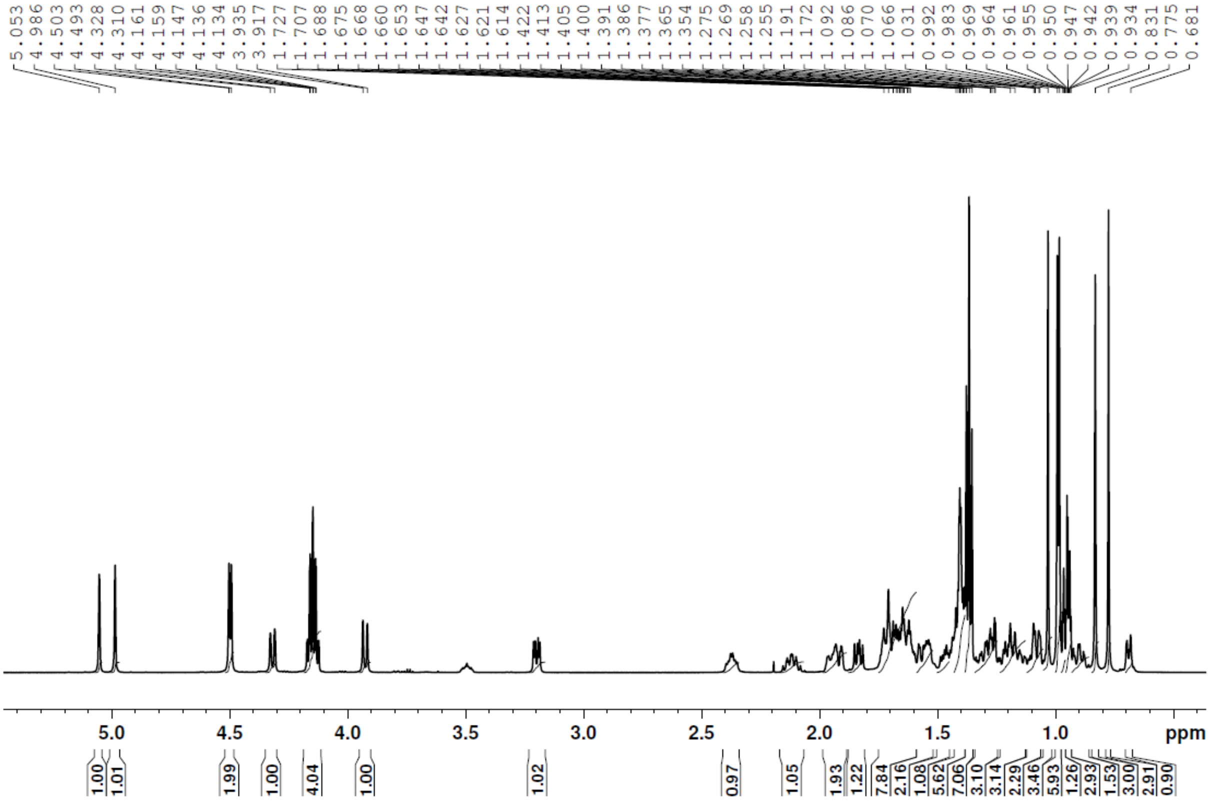

Fig. S5b. ^{13}C NMR, 28-(2-cyclopropylpropynoyl)-30-diethoxyphosphoryloxybetulin **7c**

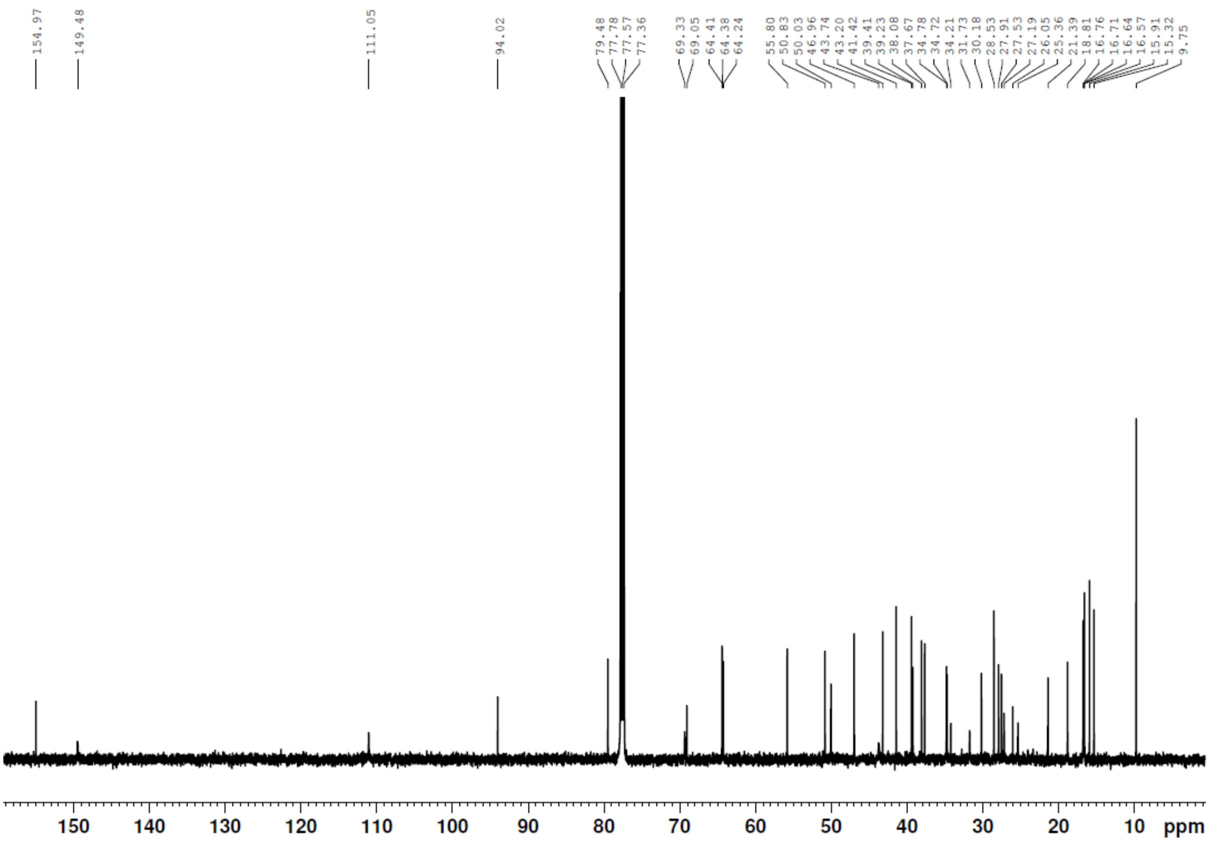

Fig. S5c. ^{31}P NMR, 28-(2-cyclopropylpropynoyl)-30-diethoxyphosphoryloxybetulin **7c**

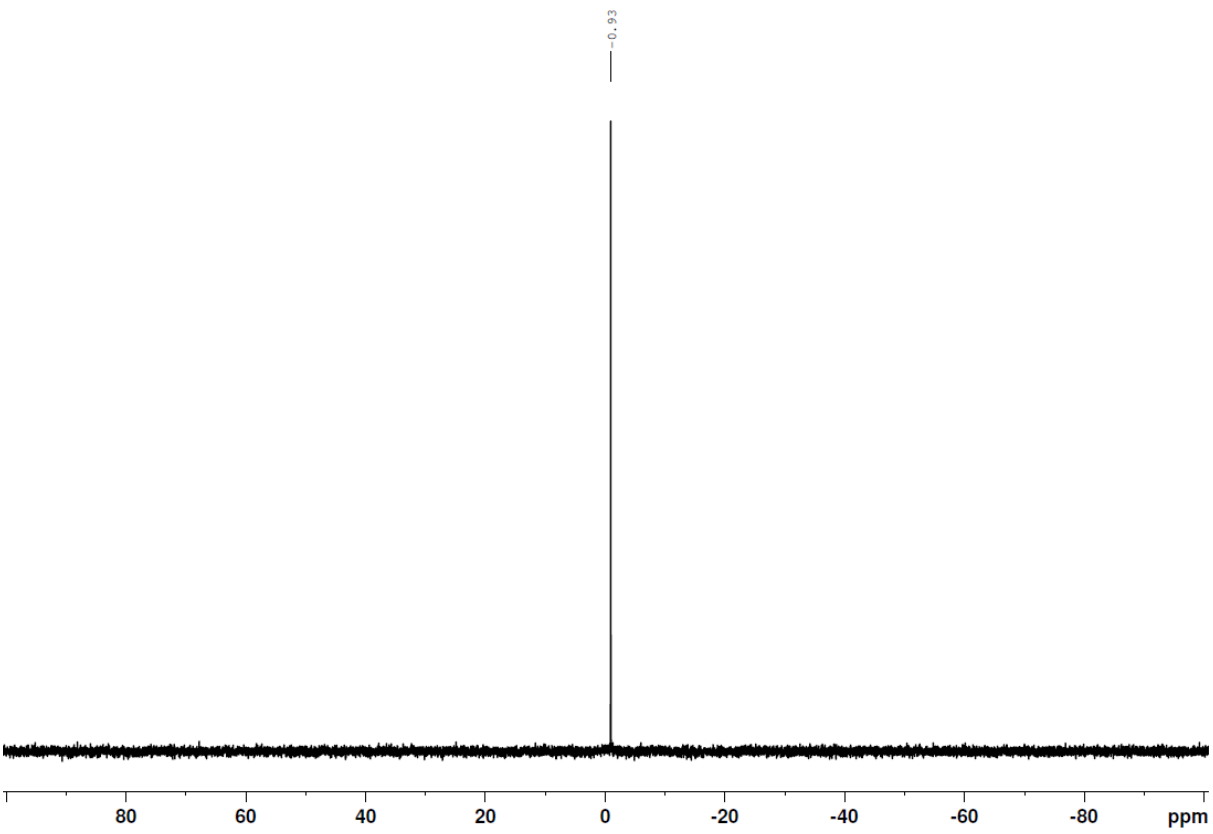

Fig. S5d. HR-MS, 28-(2-cyclopropylpropynoyl)-30-diethoxyphosphoryloxybetulin **7c**

Mass Spectrum List Report

Analysis Info

Analysis Name	D:\Data\PM_20171204\ECh 184B.d	Acquisition Date	5/29/2018 12:17:29 PM
Method	low_mass.m	Operator	KM
Sample Name	AN 40_18	Instrument	impact II 1825265.10082
Comment			

Acquisition Parameter

Source Type	APCI	Ion Polarity	Negative	Set Nebulizer	2.0 Bar
Focus	Active	Set Capillary	4000 V	Set Dry Heater	200 °C
Scan Begin	100 m/z	Set End Plate Offset	-500 V	Set Dry Gas	5.0 l/min
Scan End	2000 m/z	Set Charging Voltage	2000 V	Set Divert Valve	Source
		Set Corona	2000 nA	Set APCI Heater	450 °C

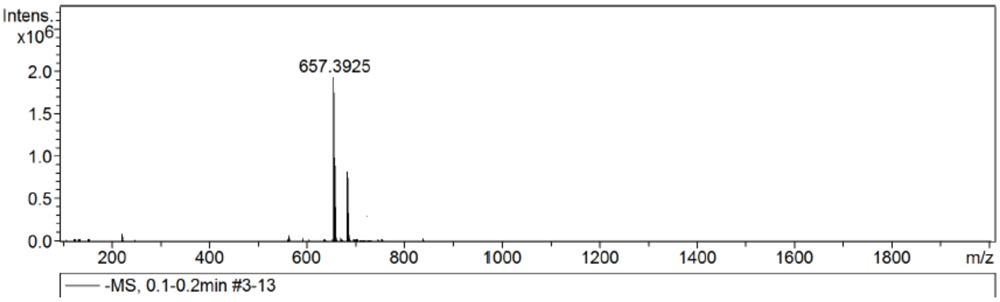

#	m/z	Res.	S/N	I	I %	FWHM
1	657.3925	35852	37519.9	1938661	100.0	0.0183
2	685.4229	46227	15393.9	830665	42.8	0.0148

Table S1. Calculated Raman band frequencies and assignments for compounds **10** and **5**.

Calculated spectra		
**5**	**10**	
3621, 3611	3640, 3625	OH stretching
3152, 3075	3077	=C-H stretching in side chain
3053-3042	3065	-C-H stretching in cycloalkane ring
3041-2834	3048-2844	CH_3, CH_2, CH stretching in side chain and cycloalkane ring
1677	1609	C=C stretching in allyl/vinyl chains
1470-1405		CH_3, CH_2, CH bending in side chain and cycloalkane ring
1386-1353		CH_3, CH_2, CH, OH bending in side chain, P=O bending in side chain
1248	-	P-O stretching in O=P-O-C moiety
-	1208	P-C stretching in O=P-C=C moiety
-	1180	P=O bending in O=P-C=C moiety
1153	-	P=O bending in O=P-O-C moiety
1100-1050		CH_3, CH_2, CH, OH bending in cycloalkane ring and side chain
1026	-	P-O bending, C-C stretching in side chain and in cycloalkane ring

-	809	P-C, CH_3, CH_2 bending, C-C stretching
776, 702	750, 765, 688	CH_3, CH_2, CH bending in side chain and cycloalkane ring, C-C stretching
660-540	660-560	C-C in cycloalkane ring
527, 470, 447, 360	567, 509	C-C stretching, CH_3, CH_2 in side chain
401	-	P-O bending in O=P-O-C
277	234	O-H bending

X-ray diffraction experiment

Colorless single crystals of good quality were preselected under a polarized light microscope. X-ray experiment was performed at 100K. The data for compound **5** were collected using an SuperNova diffractometer (Agilent Technologies) with Atlas detector. Accurate cell parameters were determined and refined using CrysAlis CCD program [57]. For the integration of the collected data, the CrysAlis RED program [58] was used.

Refinement

The crystal structure was determined using the using direct method with SHELXS-2014 [59] program and then the solutions were refined using SHELXL-2014 [59] program. H atoms were treated as riding atoms in geometrically idealized positions, fixing the C-H bond lengths at 0.95, 1.00, 0.99, 0.95 and 0.98 Å for aldehyde CH, methine CH, methylene CH_2, terminal methylene CH_2 and methyl CH_3 atoms respectively, and with Uiso(H) ¼ 1.5Ueq(C) for methyl H atoms or 1.2Ueq(C) otherwise. Hydrogen atoms involved in H-bonding (acetylenic and hydroxyl H atoms) were introduced into the calculated positions and then refined freely with isotropic atomic displacement parameters.

Crystal structure was deposited at the Cambridge Crystallographic Data Center, with deposit number CCDC 2039827 and is available free of charge via www.ccdc.cam.ac.uk/data_request/cif.

References

59. Sheldrick, G. A short history of SHELX, *Acta Cryst.* A 64, **2008**,112–122, https://doi.org/10.1107/S0108767307043930.
60. CrysAlis PRO. Agilent Technologies Ltd, Yarnton, Oxfordshire, England. 2014.
61. Sheldrick, G.M. Crystal structure refinement with SHELXL, *Acta Cryst* C71 **2015**, 3e8.

Table S2 Crystal parameters, data collection and refinement details for compound **5**

Data collection	
Diffractometer	SuperNova diffractometer (Agilent Technologies) with Atlas detector
Absorption correction	Multi-scan<br>CrysAlis PRO 1.171.38.41q (Rigaku Oxford Diffraction, 2015) Empirical absorption correction using spherical harmonics, implemented in SCALE3 ABSPACK scaling algorithm
T_{min}, T_{max}	0.592, 1.000
No. of measured, independent and observed [$I > 2\sigma(I)$] reflections	573037, 23993, 7243
R_{int}	0.0462
$(\sin \theta/\lambda)_{max}$ (Å^{-1})	0.711
Refinement	
$R[F^2 > 2\sigma(F^2)]$, $wR(F^2)$, S	0.0496, 0.1260, 1.019
No. of reflections	6602
No. of parameters	379
H-atom treatment	H-atom parameters constrained
$\Delta\rangle_{max}$, $\Delta\rangle_{min}$ (e Å^{-3})	0.66, -0.29

Absolute structure	Flack x determined using 4515 quotients [(I+)-(I-)]/[(I+)+(I-)] (Parsons, Flack and Wagner, Acta Cryst. B69 (2013) 249-259).

Table S3 Monocrystal characteristic for compound **5.**

Compound	**5**
Chemical formula	$C_{34}H_{59}O_6P$
M_r	594.78
Solvent	CH_3CN
Crystal system, space group	Monoclinic, P 21
Temperature (K)	100
a, *b*, *c* (Å)	7.3147(3); 11.8986(5); 18.6947(8)
α, β, γ [°]	90; 97.977(4); 90
V (Å^3)	1611.34(12)
Z	2
Radiation type	Cu *K*α
μ (mm^{-1})	0.128

Table S4. Selected hydrogen bonds in the crystal structure of compound **5**

D: donor, A: acceptor. Distances DH, HA, DA are in Å and DHA angles are in degrees.

Nr	D-H…A	D-H [Å]	H…A [Å]	D…A [Å]	<(DHA)	Symmetry codes
1	O1-H1…O2	0.84(5)	1.95(5)	2.786(4)	170(5)	*-x+1,y-1/2,-z*
2	O2-H2…O4	0.90(5)	1.86(5)	2.754(4)	170(5)	*-x+2,y+1/2,-z+1*
3	C30-H30B…O4	1.00(4)	2.44(5)	2.948(5)	111(3)	---
4	C31-H31B…O1	1.01(5)	2.48(5)	3.008(5)	112(3)	*-x+2,y-1/2,-z*
5	C32-H32A…O4	1.01(6)	2.62(6)	3.486(6)	144(4)	*-x+2,y-1/2,-z+1*

Chromatographic procedure by RP-TLC

The experimental lipophilicity parameters was determined using reversed-phase thin-layer chromatography (RP-TLC). The stationary phase was an aluminium backed silica gel RP-18 F_{254} S plates (Merck, Darmstadt, Germany). Chloroform solutions of reference compounds

(acetanilide, 4-bromoacetophenone, benzophenone, anthracene, DDT) and triterpenes **3-5**, **6a-6e** and **7a-7e** were spotted on to the plates in the amount of 2 μL. The chromatograms were performed in triplicate for each compound. The chromatographic chambers were filled of mobile phases consisting of a mixture of acetone (POCh, Gliwice, Poland) and aqueous solution of buffer Tris [(tris-hydroxymethyl)aminomethane] (Fluka, Loughborough, 0.2 M, pH 7.4). The percent acetone volume was varied within the range of 90-60%, in 5% increments. The spots of compounds were visualized by spraying the plates with 10% ethanolic solution of sulphuric acid (VI) and heating for 3 min at 110 ℃ [60]]. The R_M values were calculated by the equation R_M = log (1/R_f-1) on the basis of average R_f values. The R_{M0} parameters were obtained by extrapolating on the acetone content to zero, according to the equation $R_M = R_{M0} + bC$ (C – the percentage concentration of acetone in the mobile phase, b – the slope of the regression plot). The experimental lipophilicity as log P_{TLC} for triterpenes **3-5**, **6a-6e** and **7a-7e** was calculated by calibration equation

$$\log P_{TLC} = 1{,}2649R_{M0} + 0{,}2094\ (r = 0.996; SD = 0.445)$$

obtained on the correlation between the literature log P_{lit} values and the experimental R_{M0} values for standard compounds.

Table S5. The theoretical values of lipophilicity for tested compounds **3-5**, **6a-6e** and **7a-7e**.

Compound	ALOGPs	AC logP	miLogP	ALOGP	MLOGP	XLOGP2	XLOGP3
**3**	5.50	5.86	7.33	5.98	5.83	7.84	8.18
**4**	7.00	6.24	7.97	6.68	6.61	8.49	8.89
**5**	5.33	5.26	5.56	5.93	5.97	7.01	7.74
**6a**	6.23	5.15	7.87	9.23	6.79	9.04	9.85
**7a**	5.78	5.21	7.21	7.58	6.37	8.03	8.8
**6b**	7.32	7.19	8.91	9.13	7.13	10.03	10.87
**7b**	6.47	6.23	8.03	7.53	6.55	8.41	9.31
**6c**	6.89	7.73	9.25	9.8	7.78	10.86	11.97
**7c**	6.23	6.49	8.42	7.86	6.89	8.83	9.85
**6d**	7.80	10.10	9.44	11.19	8.28	13.24	13.40
**7d**	6.95	7.68	8.63	8.56	7.15	10.13	10.57
**6e**	6.64	6.54	7.81	10.35	6.47	9.37	9.93
**7e**	6.17	5.90	7.19	8.14	6.21	8.19	8.84

Table S6. The correlation matrix for theoretically obtained lipophilicity parameters of compounds **3-5, 6a-6e** and **7a-7e**.

	ALOGPs	AC logP	miLogP	ALOGP	MLOGP	XLOGP2	XLOGP3
**ALOGPs**	1.000	0.825	0.889	0.814	0.889	0.888	0.884
**AC logP**		1.000	0.812	0.725	0.877	0.939	0.907

**miLogP**	1.000	0.784	0.904	0.889	0.909
**ALOGP**		1.000	0.840	0.884	0.903
**MLOGP**			1.000	0.959	0.925
**XLOGP2**				1.000	0.987
**XLOGP3**					1.000

62. Bębenek, E.; Bober-Majnusz, K.; Siudak, S.; Chrobak, E.; Kadela-Tomanek, M.; Wietrzyk, J.; Boryczka, S. Application of TLC to evaluate the lipophilicity of newly synthesized betulin derivatives. *J Chromatogr Sci* **2020**, 58, 323-333. https://doi.org/10.1093/chromsci/bmz117.
